# Genomic Sequence of Canadian *Chenopodium berlandieri*: A North American Wild Relative of Quinoa

**DOI:** 10.3390/plants12030467

**Published:** 2023-01-19

**Authors:** Mark E. Samuels, Cassandra Lapointe, Sara Halwas, Anne C. Worley

**Affiliations:** 1 Centre de Recherche du CHU Ste-Justine, Montréal, QC H3T 1C5, Canada; 2 Département de Biochimie, Université de Montréal, Montréal, QC H3T 1C5, Canada; 3 Département de Médecine, Université de Montréal, Montréal, QC H3T 1C5, Canada; 4 Department of Anthropology, University of Manitoba, Winnipeg, MB R3T 2M8, Canada; 5 Department of Biological Sciences, University of Manitoba, Winnipeg, MB R3T 2M8, Canada

**Keywords:** *Chenopodium berlandieri*, pitseed goosefoot, *Chenopodium quinoa*, DNA barcoding, genome sequencing, genome assembly, wild crop relatives, *SOS1*

## Abstract

*Chenopodium berlandieri* (pitseed goosefoot) is a widespread native North American plant, which was cultivated and consumed by indigenous peoples prior to the arrival of European colonists. *Chenopodium berlandieri* is closely related to, and freely hybridizes with the domesticated South American food crop *C. quinoa*. As such it is a potential source of wild germplasm for breeding with *C. quinoa*, for improved quinoa production in North America. The *C. berlandieri* genome sequence could also be a useful source of information for improving quinoa adaptation. To this end, we first optimized barcode markers in two chloroplast genes, *rbcL* and *matK*. Together these markers can distinguish *C. berlandieri* from the morphologically similar Eurasian invasive *C. album* (lamb’s quarters). Second, we performed whole genome sequencing and preliminary assembly of a *C. berlandieri* accession collected in Manitoba, Canada. Our assembly, while fragmented, is consistent with the expected allotetraploid structure containing diploid *Chenopodium* sub-genomes A and B. The genome of our accession is highly homozygous, with only one variant site per 3–4000 bases in non-repetitive sequences. This is consistent with predominant self-fertilization. As previously reported for the genome of a partly domesticated Mexican accession of *C. berlandieri*, our genome assembly is similar to that of *C. quinoa.* Somewhat unexpectedly, the genome of our accession had almost as many variant sites when compared to the Mexican *C. berlandieri*, as compared to *C. quinoa*. Despite the overall similarity of our genome sequence to that of *C. quinoa*, there are differences in genes known to be involved in the domestication or genetics of other food crops. In one example, our genome assembly appears to lack one functional copy of the *SOS1* (salt overly sensitive 1) gene. *SOS1* is involved in soil salinity tolerance, and by extension may be relevant to the adaptation of *C. berlandieri* to the wet climate of the Canadian region where it was collected. Our genome assembly will be a useful tool for the improved cultivation of quinoa in North America.

## 1. Introduction

Adaptation of quinoa for large-scale farming poses significant challenges outside South America. One such challenge is limited international access to most quinoa germplasms [1]. Moreover, domesticated landraces of quinoa are the result of extensive selection for optimized productivity in local conditions. Such landraces may not be useful for adapting quinoa to widely different local conditions elsewhere in the world, even when access to their germplasms is available.

### 1.1. Wild Crop Relatives of Quinoa

An approach to addressing germplasm limitations for other agricultural crops is to utilize wild relatives as sources of genetic diversity [2,3]. In the case of quinoa, the immediate wild relative is also in South America (*C. hircinum* or a related species) [4]. Genomic studies indicate that quinoa is probably derived originally from North America [4]. The native North American plant *Chenopodium berlandieri*, also known as pitseed goosefoot, is very similar to quinoa, suggesting a recent common ancestor [4]. Both *C. quinoa* and *C. berlandieri* are allotetraploid, consisting of two sub-genomes A and B, that resemble the genomes of diploid *C. pallidicaule* and *C. suecicum*, respectively [4]. *Chenopodium berlandieri* and *C. quinoa* are interfertile, indicating that *C. berlandieri* should be useful as a wild crop relative of quinoa, as it is adapted for growth in various local conditions.

### 1.2. Historical Cultivation of C. berlandieri

*C. berlandieri* is itself of interest as a potential food crop. It was cultivated in North America prior to European colonization, as part of the Eastern Agricultural Complex (EAC) [5,6,7]. Seeds of *C. berlandieri* found in cooking pits at some archaeological sites, including in Canada, are larger than seeds found in wild plants today, and have thinner testa [7]. These observations indicate that *C. berlandieri* was not just cultivated but at least partially domesticated, thousands of years ago. Given that *C. berlandieri* is native to Canada, and that *C. berlandieri* appears to share the nutritional advantages of quinoa [8], the idea of re-domesticating it for cultivation is intriguing. A similar idea has been proposed for the diploid native North American *C. ficifolium* [9].

### 1.3. Adaptation of C. berlandieri in Canada

Growth of *C. berlandieri* in Canada has undoubtedly involved adaptations to short growing seasons and cold winters due to the northern latitudes, as well as varying moisture levels across the species range. For example, winter lows in regions where *C. berlandieri* has been reported, vary from −10 °C in southern Quebec to −40 °C in southern Manitoba. Precipitation during the growing season in 2022 ranged from 150 mm in southern Saskatchewan (home of the commercial quinoa producer Norquin) to more than 400 mm in southern regions of Manitoba, Ontario, and Quebec (Agriculture and Agri-Food Canada https://www.agr.gc.ca/DW-GS/historical-historiques.jspx (accessed on 6 January 2023)). Comparison of a Canadian *C. berlandieri* genome to that of *C. quinoa* should yield candidate genetic variants present in *C. berlandieri*, that could be introduced into quinoa to improve its local adaptation.

### 1.4. Utility of C. berlandieri Genome Sequencing

Whether as a source of genetic information or to initiate a de novo program of domestication, a genome assembly of wild *C. berlandieri* would be extremely helpful. A genome of *C. berlandieri* subspecies *nuttaliae* has been reported [10]. That accession is from Mexico, is at least partly domesticated, and its similarity to *C. berlandieri* from more northern regions is uncertain. Raw genomic sequence reads of other *C. berlandieri* accessions are in the KAUST (King Abdullah University of Science and Technology) *Chenopodium* database (https://www.cbrc.kaust.edu.sa/chenopodiumdb/ (accessed on 6 January 2023)). However, there has been no published assembly or analysis of a wild *C. berlandieri* genome, and no sequencing of plants of Canadian provenance.

### 1.5. Field Identification of C. berlandieri

An immediate issue with the study of *C. berlandieri* is its identification. Distinguishing members of the *Chenopodium* species complex in the field is challenging, in particular for *C. berlandieri* and the Eurasian invasive *C. album* (lamb’s quarters). This is a significant problem in Canada, where *C. berlandieri* is native [11], but where *C. album* was introduced by the Europeans, and is now widely distributed [12]. These two species are almost indistinguishable by overall appearance, but can be discriminated by the morphology of mature seeds late in the season [13,14]. DNA sequence markers to study the overall phylogeny of *Chenopodium* species have been developed, although these are not optimized to discriminate among the species found in Canada [15,16,17]. Devi et al. reported sites in the *rbcL* and *matK* genes that distinguished *C. quinoa* from *C. album*, but they did not report the status of *C. berlandieri*, and their primers were designed for gel-based, not sequence-based analyses [18]. However, their success bodes well for the utility of these genes for *Chenopodium* species identification.

### 1.6. Study Objectives

The main objective of our study was to generate a preliminary whole genome assembly of Canadian *C. berlandieri*, and to compare it to other sequenced *Chenopodium* genomes including domesticated *C. quinoa*, and the two diploids *C. pallidicaule* and *C. suecicum*. As an ancillary objective, we sought to optimize barcode markers that could discriminate between *C. berlandieri* and *C. album*. We report the development of useful barcode markers, and the assembly and analysis of the first Canadian *C. berlandieri* genome sequence.

## 2. Results

### 2.1. Collections

**Figure 1:**
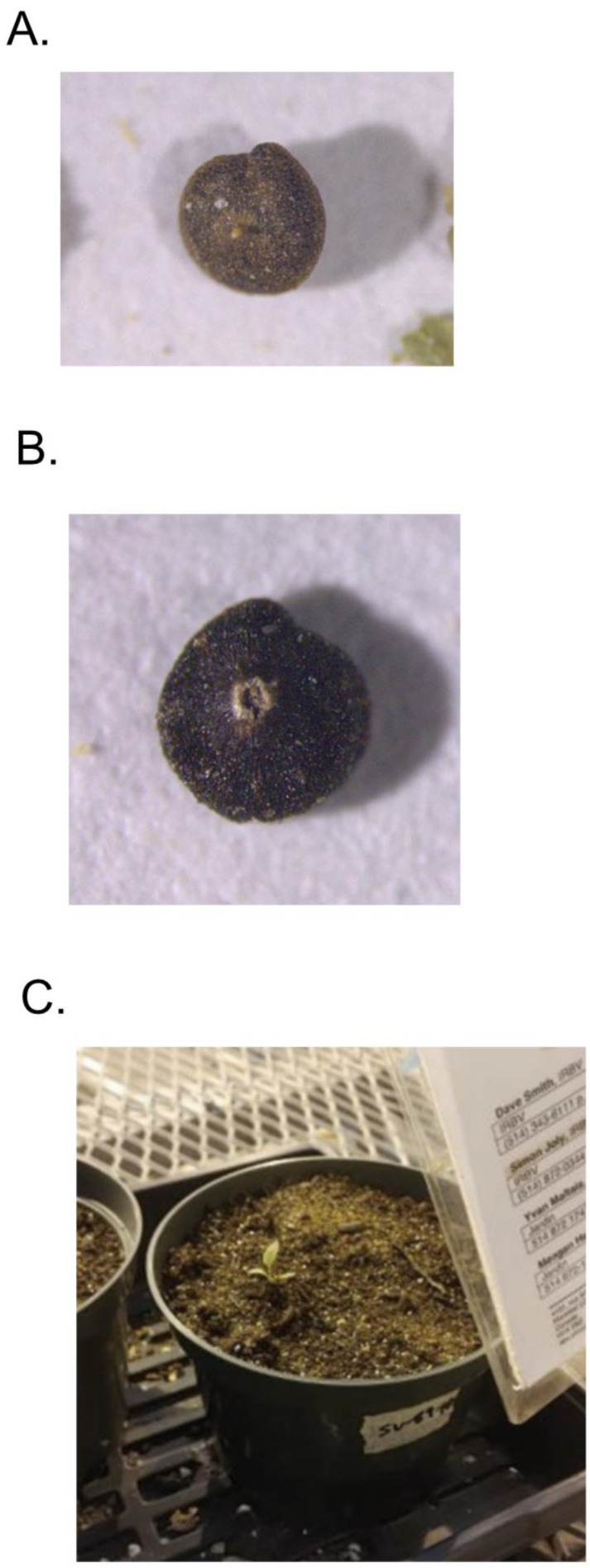
Seeds and plants from SV site in Manitoba. (**A**) seed from plant SV89-10. (**B**) seed from plant SV92-10. (**C**) plant from seed of plant SV89-10, at three weeks post-germination.

Plants were grown from seeds collected at the SV and AB sites in Manitoba, Canada, near Winnipeg [8,19]. The seeds showed a distinctive pitted pattern underneath the pericarp (Figure 1). As positive controls, seeds from various *Chenopodium* species accessions including *C. berlandieri, C. quinoa* and *C. album* were obtained from the Agricultural Research Station of the U.S. Department of Agriculture in Iowa, germinated, and grown to obtain leaf material for DNA extraction. There are concerns about the correctness of species identification of some *Chenopodium* accessions in the repository, but we chose accessions that are well-documented from known contributors. The leaf and plant morphology of the *C. berlandieri* plants from Manitoba and from the ARS accession were indistinguishable from each other and from the *C. album* plants. *C. quinoa* was grown from commercially sold seeds (West Coast Seeds, Vancouver, BC, Canada).

### 2.2. Barcoding

The literature on the use of DNA barcoding to explore the phylogeny of *Chenopodia* has preferentially emphasized the maximal breadth of utility across the entire genus [17,18,20,21]. This does not necessarily lead to the optimization of the markers for the species of interest here. For our specific purpose of distinguishing *C. berlandieri* from *C. album*, sequences of the *matK* and *rbcL* genes of *C. quinoa* and *C. album* were compared, derived from recently published chloroplast sequences for the two species [22]. *C. quinoa* was used initially, as full chloroplast sequences from *C. berlandieri* were not available, and these two species are expected to be identical or nearly so in their chloroplast genomes. Unpublished *C. berlandieri* chloroplast sequences were also generously provided by D. Jarvis. Partial sequences for these genes in many *Chenopodium* species are available in the International Barcode of Life database (iBOLD, http://v4.boldsystems.org/ (accessed on 6 January 2023)). There are many discrepancies and/or gaps in individual sequences from the same species in the database, thus these were used for further verification but not the development of amplicons. We designed and tested multiple primer pairs, with the criteria that the primer sites be conserved in the two species *C. quinoa* and *C. album*, the amplicons be of reasonable size for Sanger sequencing, and the amplicons include sites different in the two species.

#### 2.2.1. *rbcL* Barcoding

Many published studies of plant barcoding employing the *rbcL* gene use a sub-region of the gene, referred to as *rbcLa* [23,24]. There are no sequence differences between *C. quinoa* and *C. album* in that region of the gene, therefore we designed a novel amplicon (Figure S1A,B). In our amplicon rbcLM1, which is primarily downstream of *rbcLa* in the gene, there are two differences between the *C. quinoa* and *C. album* published sequences. As seen in Figure S2, commercial quinoa, USDA *C. berlandieri*, and plant SV89-10 from the SV site in Manitoba all had the *C. quinoa* sequences at both sites in the rbcLM1 amplicon.

#### 2.2.2. *matK* Barcoding

Pairwise alignment of the *matK* genes from *C. quinoa* and *C. album* showed more differences than with *rbcL*; this is consistent with published barcoding studies that find *matK* to be more variable than *rbcL* [25]. We designed several different novel PCR amplicons as well as testing some from the literature and iBOLD database, using the same criteria as for *rbcL*. We had success with different amplicons, but for the study, we used the primer pair matK390-F [26] and matK3FK1M-R (iBOLD, submitted by M. Kuzmina). In this PCR amplicon, there are six differences between *C. quinoa* and *C. album*, of which five are easily observed in clean sequence traces, the sixth being too close to one primer (Figure S3A,B). As shown in Figure S4, commercial quinoa, USDA-derived *C. berlandieri*, and the Manitoba SV89-10 plant have the *C. quinoa* sequence at all five sites. There was one additional difference in the USDA-derived *C. berlandieri*, which was not shared by the NCBI reference sequence or the Manitoba SV plant.

Our results with both genes *rbcL* and *matK* are consistent with the Manitoba plant identification as *C. berlandieri*, and that *C. quinoa* and *C. berlandieri* are almost or fully identical in these gene sequences.

### 2.3. Genome Sequencing and Assembly

Having confirmed the species identification of Manitoba plant SV89-10, we submitted DNA for Illumina 2 × 151 bp paired-end sequencing. Given the availability of chromosome-level assemblies of *C. quinoa* and *C. berlandieri nuttaliae*, as well as less complete assemblies of *C. pallidicaule* and *C. suecicum*, paired-end Illumina sequencing of a short insert library was considered sufficient to compare SV89-10 with the other *Chenopodium* species.

#### 2.3.1. Total Sequence Obtained

We obtained 96.9 Gbp of raw total sequence, or approximately 70-fold coverage assuming the same genome size of 1.4 Gb as *C. quinoa*. FastP was used to clean the reads, restricted to the removal of adapters and poly-G homopolymer runs. The total sequence post-trimming was 95.3 Gb (estimated 68-fold genome coverage), of which 94% was of quality Q30 or better.

#### 2.3.2. k-mer Analysis

**Figure 2:**
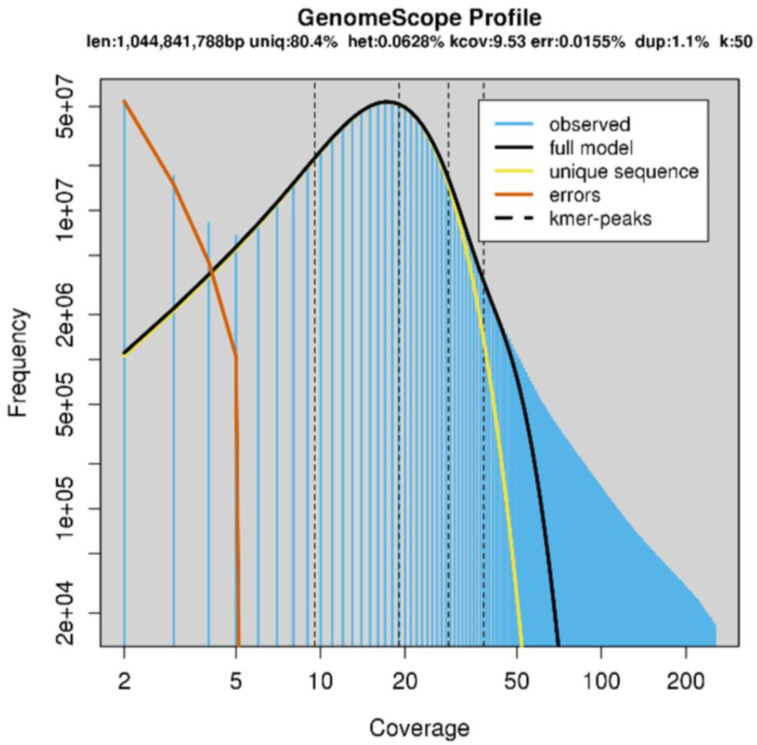
GenomeScope model with k-mer = 50, input forward reads trimmed with FastP to remove the adapter and poly-G sequences.

To confirm the expected genome size, k-mer analyses were performed using KMC3 (25-mer and 50-mer) and Jellyfish (25-mer), followed by modelling with GenomeScope. Using the output of KMC3 with only the trimmed forward reads as input, and using k = 50, GenomeScope predicted a genome size of approximately 1 Gb (Figure 2, Table 1. The presence of a significant right-side shoulder (Figure 2, black line, full model, versus yellow line, unique sequences) is presumably due to the incomplete resolution of repetitive elements and the two parental sub-genomes. This is also expected to reduce slightly the predicted genome size versus the true size. Jellyfish gave similar results using k = 25 (data not shown). The heterozygosity predicted by these programs was not considered reliable given the tetraploid genome structure and expected high repeat content.

#### 2.3.3. Genome Assembly Using Platanus

Assembling with Platanus v1, the optimal initial k-mer size was k = 96, yielding an N50 of 6.641 kb and L50 of 42,216 for the scaffolded assembly (Table 2). The total scaffold length was 1.24 Gb, of which 900 Mb is in scaffolds of 1 kb or greater. This is reasonably consistent with the expected genome size of 1.4 Gb, and indicates the minimal collapse of the two sub-genomes, or splitting of heterozygous and repetitive regions.

#### 2.3.4. Genome Assembly Using Abyss

Assembly was also performed using Abyss, with initial k-mer sizes of 80, 88, 96, 104, and 112. The optimal assembly with Abyss was at k = 104, with a scaffold N50 of 3.125 kb (shorter contigs than Platanus) and a scaffold L50 of 88,430 (more contigs than Platanus), with a total assembled length of 1.31 Gb (longer than Platanus) (Table S1). Comparing the assembly of individual genes with Platanus versus Abyss; in most cases, all coding exons for genes could be found, usually in multiple fragmented scaffolds (see below). The specific exon fragmentation patterns were not always the same with the two assembly programs for the same gene.

#### 2.3.5. Repetitive Sequence Content of Platanus Assembly

Subsequent analyses were based on the Platanus k-mer 96 assembly. Repetitive sequence content was assessed with RepeatMasker, using a set of 1672 quinoa repetitive sequences annotated in the PlantRep database [27]. A total of 40% of the assembled genome consists of identified repetitive elements, mostly of the LTR type (primarily Gypsy and Ty1/Copia) with a small percentage of DNA transposon and LINE elements (Table 3). An additional 20% of the genome was annotated as unclassified repeats, whose nature is not clear in the RepeatMasker report. The published *C. quinoa* version 1 genome repetitive sequence content was almost identical to that of our assembly, in terms of the percent of the genome in the various repeat classes (including the unclassified repeats) [4]. Repeat content statistics are not available on the CoGe website (https://genomevolution.org/coge/ (accessed on 6 January 2023)) for the *C. quinoa* version 2 genome assembly.

#### 2.3.6. BUSCO Analysis of Genome Completion

The Platanus k96 assembly was analyzed for completion with BUSCO, using a set of standardized eudicot genes (Table 4). Of 2326 genes searched by BUSCO in its internal gene set, 1859 were found complete in either one (645) or two (1214) copies, 181 were fragmented, and 286 (12%) were not found. We manually checked several genes reported as missing by BUSCO, by manually BLASTING the assembly. We found homologous sequences in each case, in very small contigs. Thus, some genes were probably treated as missing by BUSCO due to their excessive fragmentation. The BUSCO result is consistent with a nearly complete genome assembly, at least for gene-containing regions. For comparison, BUSCO analysis of the quinoa version 1 genome observed complete genes equally split between one and two copies [4], suggesting that our assembly did a better job of resolving the two sub-genome homologs for many of the BUSCO genes (BUSCO analysis is not available for the *C. quinoa* version 2 genome assembly). The *C. quinoa* version 1 assembly was only missing 2.7% of BUSCO genes, versus the 12% missing in our assembly. In the *C. quinoa* analysis, only 936 genes were searched versus 2326 genes in the BUSCO database version we employed. Our larger gene search set may contain sequences more distant from *Chenopodium* species, hence harder for BUSCO to identify. By comparison to our Platanus assembly, BUSCO analysis of our best Abyss assembly had fewer two-copy genes and more fragmented and missing genes (Table S2).

#### 2.3.7. Heterozygosity Analysis of Platanus Assembly

To obtain a sense of the genetic variation in our sequenced accession, the heterozygosity of the assembly was assessed by aligning the trimmed individual sequence reads back to the Platanus-assembled scaffolds. Using the default stringency quality score filtering, there were 1.38 million SNPs called, approximately one SNP every kilobase, with a Ts/Tv ratio of 1.53. On visual inspection, it was observed that heterozygous sites were not distributed equally. As seen in Figure S5, many long scaffolds contained no heterozygous sites at all, others had one or two, and there were some regions with many clustered heterozygous sites in regions of higher-than-average coverage. Regions with higher-than-average coverage are likely to involve repetitive sequences, for which misalignments of short reads are more likely. When variant sites were filtered to exclude regions identified as repetitive by RepeatMasker, only 394,190 heterozygous SNPs were retained, fewer than one-third of all called SNPs and an average of one SNP every 3000–4000 bp. The Ts/Tv ratio of repeat masked SNPs was 1.44.

In addition to heterozygous variant sites in the assembly, there were 12,091 SNPs called homozygotes. In principle, there should not be any since the variant calling was done with sequence reads aligned to the genome assembled from those reads. Presumably, this small number of homozygous calls resulted from slight differences in the assembly and variant calling algorithms or stringency parameters. We also noted that some retained heterozygous sites were immediately adjacent to regions marked as repetitive; these SNPs might potentially also result from repeat read misalignment due to the adjacent repeats, or possibly be in repeats not fully masked at the borders by RepeatMasker.

#### 2.3.8. Genomic Variation versus Other Tetraploid Chenopodium Genomes

We assessed the extent of variation with respect to other tetraploid *Chenopodium* genomes by aligning our trimmed sequence reads to the assembled genomes of *C. quinoa* (version 2) and *C. berlandieri nuttaliae* (downloaded from the CoGe database), using the same variant calling pipeline as above. Focusing on SNPs only, in regions identified by RepeatMasker as non-repetitive in the other genomes, there were 2,616,323 homozygous and 1,361,174 heterozygous variant sites called for the *C. quinoa* genome, and 2,433,258 homozygous and 972,698 heterozygous variant sites called for the *C. berlandieri nuttalliae* genome.

### 2.4. Organelle Genome Assembly

When DNA for genome sequencing is obtained from total leaf material, chloroplast, and mitochondrial genomes will be present in the raw sequence data. In order to assemble the organelle genomes, Platanus was run on the trimmed sequence files with a minimum k-mer coverage of 200. This was expected to simplify the assembly, assuming that there would be a significant excess of reads derived from the organelle genomes compared to nuclear genomes (although some nuclear repetitive sequences would also be expected in this reduced set of input sequence reads).

#### 2.4.1. Chloroplast Genome

The chloroplast single copy genome was recovered in two large scaffolds (Figure S6): scaffold1316 (quinoa chloroplast bp 1–83,656), and scaffold1317 (quinoa chloroplast bp 108,719–126,754), both with nearly 100% identity to the *C. quinoa* chloroplast, plus one very small scaffold1318 (quinoa chloroplast bp 126,754–126,962). Additional scaffolds aligned to the chloroplast genome inverted repeat regions (data not shown). The quinoa *matK* gene aligned to chloroplast scaffold1316 across the entire protein coding region with a single mismatch; similarly, the quinoa *rbcL* gene aligned to scaffold1316 across the entire protein coding region with no mismatches (data not shown). In the main genome assembly with all k-mer coverages included, the chloroplast genome was recovered in many fragmented scaffolds; it is unclear why these were not collapsed into the same large scaffolds as with the organelle-specific assembly.

#### 2.4.2. Mitochondrion Genome

Much of the mitochondrial genome was recovered in two large scaffolds (Figure S7): scaffold457 (quinoa mitochondrion bp 1–46,824; 48,033–129,563; 129,639–183,927; 236,316–236,674; 303,647–315,003), and scaffold544 (quinoa mitochondrion bp 190,638–235,545). It is unclear why the larger of the two scaffolds contains multiple internal rearrangements, but mitochondrial DNA variation of this type is known in angiosperms (T. Sharbel, pers. comm.). Again, alignments of these scaffolds to the *C. quinoa* mitochondrial genome were almost 100% identical, discounting the internal rearrangements. The remainder of the mitochondrial genome (approximately quinoa mitochondrion bp 237,000–303,000) was not present in the organelle-specific assembly. Many scaffolds were recovered by BLASTing this segment of the quinoa mitochondrial genome to the whole genome assembly, but multiple mismatches were observed in the longer scaffolds. Possibly the mitochondrial genomes are too heterogeneous in this region to assemble using the specific mismatch parameters, or else multiple varying copies of some mitochondrial sequences might be present in nuclear DNA.

Surprisingly, scaffold1316 of the chloroplast assembly also had BLAST hits to the quinoa mitochondrial genome. Therefore, we compared the *C. quinoa* chloroplast and mitochondrial genomes, and observed multiple well-aligned, short segments (Figure S8), the longest being an almost 2 kb segment containing the *pbsA* gene and a fragment of the *matK* gene, neither of which gene is annotated in the mitochondrial genome in the NCBI nucleotide database. A direct alignment of the *C. quinoa matK* gene to the *C. quinoa* mitochondrial genome also showed a 257 bp, 100% alignment. Fortunately, the *matK* barcoding PCR amplicon we employed has minimal overlap with the fragment of the *matK* gene in the mitochondrial genome assembly, thus the mitochondrial sequence would not contribute to the chloroplast barcode sequencing amplicon.

The organelle genome scaffolds are included in our assembly as submitted to the CoGe archive.

### 2.5. Comparative Genomics of Agriculturally Relevant Genes

A comprehensive analysis of all genes believed to play a part in regulating agriculturally important traits (seed number, seed weight, seed coat thickness, germination rate, inflorescence morphology, photoperiod, etc.) is beyond the scope of this study. We looked at several genes to assess the utility of comparing their structure in our assembly to other *Chenopodium* species. We looked at putative orthologs (hereafter referred to generally as homologs, as true functional equivalence is typically uncertain) in the other *Chenopodium* species for which gene models are available in CoGe, specifically *C. quinoa* version 2, *C. berlandieri nuttaliae* from Mexico, *C. suecicum,* and *C. pallidicaule*. *C. pallidicaule* is a diploid species very similar to the A sub-genome of the allotetraploids *C. quinoa* and *C. berlandieri*, while the annotated *C. suecicum* genome in CoGe is that of a diploid species very similar to the B sub-genome of the allotetraploids [4]. Note that there are other plants also identified as *C. suecicum*, which are reportedly hexaploid [28].

#### 2.5.1. *SOS1*

The first gene we examined was *SOS1*, a gene implicated in tolerance to growth in high salinity soil, and hence of considerable importance and currently under study in many plant species including *C. quinoa* [29,30,31]. The *C. quinoa* and *C. berlandieri nuttaliae* genomes both contain two *SOS1* genes, on homologous chromosomes C6A and C6B. In our *C. berlandieri* assembly, sequences homologous to both genes were present, each fragmented in several scaffolds. Each individual scaffold had a significantly higher identity to one versus the other homolog in the other assembled genomes, and could thus be confidently assigned. It was then straightforward to manually assemble the individual scaffolds to include all annotated coding exons in complete versions of the two sub-genomic *SOS1* homologs. GMAP and Splign were used to create gene models from our assembly, using either the quinoa or *C. berlandieri nuttaliae* gene models as a reference (essentially identical results were obtained with either reference gene). The A and B sub-genome homologs were compared separately, with the *C. suecicum* homolog included with sub-genome B, and *C. pallidicaule* with sub-genome A.

As seen in Figure S9A, the B sub-genome *SOS1* homologs are extremely similar. The B homolog in our assembly is identical to that of *C. berlandieri nuttaliae* at all but two positions; there are only a handful of single amino acid differences among the four gene homologs. The major difference is the presence of four insertions in the *C. quinoa* B homolog, one of which is shared with *C. suecicum*. These all appear to result from alternative splicing. The insertion of amino acids IRIW results from using an alternative splice donor site in *C. quinoa* in exon 3, downstream of the donor site used in the equivalent *C. berlandieri nuttaliae* exon 2 (Figure S9B,C, the different exon numbering results from the software interpretation of the alternative splicing patterns). Similarly, the insertion of CDLTSLNLT in the *C. quinoa* homolog results from the use of a downstream splice donor site in exon 7, compared to the donor site in the equivalent exon 6 of *C. berlandieri nuttaliae* (Figure S9D,E). It should be noted that the two GT donor sites used in the *C. berlandieri nuttaliae,* and other species gene models are both present in the *C. quinoa* genomic sequence. The IRIW sequence is present in the *C. suecicum* gene model, whereas the CDLTSLNLT is absent.

Similar reasons were found for the other two insertions in the *C. quinoa* C6B model, the DSDEVITSV and VIVDKA sequences. In both these cases, the insertion is due to the inclusion of microexons coding these amino acids. Again, using the *C. quinoa* coding sequence as a reference, these exons are included when gene models are built with the genomic sequences of *C. berlandieri nuttaliae* or our *C. berlandieri* assembly. The exclusion of the four regions in the annotation in our genome is thus just for consistency with the *C. berlandieri nuttaliae*. Otherwise, it is unclear why these four inserted segments are differentially annotated among the database genome annotations.

With the *C. suecicum* genomic sequence, the DSDEVITSV exon is included and remains in the frame, but the VIVDKA exon, when manually inserted, includes a single nucleotide insertion in the genomic sequence that would cause a frameshift disrupting most of the protein-coding sequence (Figure S9F). 

The various *SOS1* A sub-genome homologs are more different from each other than the B sub-genome homologs, again primarily due to multiple insertions or deletions in the *C. quinoa* C6A gene versus the others (Figure S10A). As with the B homologs, if the *C. quinoa* A homolog coding sequence is used as a reference, many of the alternative splice events can be used in the genomic sequences of both *C. berlandieri* assemblies, but would create a frameshift in the *C. pallidicaule* genome assembly. Thus, as with the B sub-genome *SOS1* structures, the correct homologous gene models for some of the other species remain uncertain. Aside from the alternative splicing patterns, in the regions aligned equivalently across all the homologs the A genes appear to have more single residue differences among themselves than do the B genes.

Uncertainties about the correct exon structure notwithstanding, the C6A homolog in our *C. berlandieri* assembly appears to be inactivated due to two different point mutations. A novel stop codon in exon 17 (defined using the *C. berlandieri nuttaliae* C6A coding sequence as reference) truncates more than a quarter of the C-terminus of the protein (Figure S10B). An AG to AT change at the splice acceptor site of exon 11 nominally renders that splice site inactive (Figure S10C). Both positions in our whole genome assembly are well covered with many reads containing no heterozygous reads in the assembly, thus a technical artifact of the genome sequencing is very unlikely to explain these differences.

#### 2.5.2. HAIKU Pathway Genes

One of the key pathways in controlling seed size via endosperm development in *Arabidopsis thaliana* is the HAIKU pathway, involving the *IKU1*, *IKU2,* and *MINI3* genes [32,33,34]. All three of these genes encode transcription factors, of various protein families. We compared the protein sequences of homologous genes in the available *Chenopodium* species genomes and in our assembly.

The *Arabidopsis IKU1* gene has only a single homologous gene in the *C. suecicum* and *C. pallidicaule* genomes. These sequences were used to search the *C. quinoa* and *C. berlandieri nuttaliae* genomes. In both genomes, a pair of highly similar genes was identified on homologous chromosomes, one more similar to *C. suecicum* and the other to *C. pallidicaule* in each case. For the most part, these are all single exon genes. However, the *C. suecicum* gene model has two introns, removed by splicing to generate a protein with two internal in-frame deletions with respect to the other genes. The rationale of this alternative gene model is not obvious, but for purposes of comparing all the Chenopodium species genes, these two introns were retained in a derived *C. suecicum* model. The inclusion of the two introns had no deleterious effect on the protein-coding of the new gene model, which was highly similar to the other genes across the two otherwise deleted segments. Our *C. berlandieri* genome assembly was queried with TBLASTN using these various protein models, and only two homologous sequences were found, on different scaffolds, one more similar to the *C. pallidicaule* gene and the other to the *C. suecicum* gene. Aligning the sub-genome A-like and sub-genome B-like homologs separately, there were two noticeable differences among the various species. In *C. quinoa* sub-genome A, a slightly shorter amino terminus is encoded lacking the first 10 amino acids present in the other genes (Figure S11A). Examining the genomic sequence of this region of the *C. quinoa* chromosome, there are no other upstream ATG start codons in-frame, so this appears to be a real difference and not a gene modelling issue. Otherwise, there are only a few missense variations among the homologs, and only one between our assembly gene model and that of the *C. berlandieri nuttaliae* gene model. The B sub-genome proteins are extremely similar including the amino termini, except for differences in the length of a run of poly-asparagines starting about position 44; there are five asparagines in the *C. suecicum* and *C. quinoa* C5B genes, six in *C. berlandieri nuttaliae*, and eight in the gene predicted in our assembly (Figure S11B).

The *Arabidopsis IKU2* gene has several potential homologs in the genomes of *C. pallidicaule* and *C. suecicum*. In each case, one gene had a much greater identity. These were selected as the most likely homologs in each genome. The gene model for *C. pallidicaule* included one intron and yields a predicted protein product that aligns well with the *Arabidopsis* gene for the first 862 amino acids, and for part of the C-terminus, with an internal deletion of 71 amino acids of the *Arabidopsis* gene at the site of the intron in the *C. pallidicaule*. The missing 71 amino acid segment could not be found in any reading frame in the *C. pallidicaule* genome sequence covering the intron, but is present in many chromosomal locations by TBLASTN of the *C. pallidicaule* genome with between 50–70% identity, suggesting that it is encoded by a repetitive element that may be inserted into the *Arabidopsis* gene. The *C. pallidicaule* gene model was used as the type for the *IKU2* putative homolog in the other *Chenopodia*. In *C. suecicum*, the predicted gene model was different than the equivalent *C. pallidicaule* gene model, having a different splice pattern with a much larger intron, leading to an internal deletion of 23 amino acids relative to the *C. pallidicaule* predicted protein. Additionally, the *C. suecicum* predicted protein began at an internal methionine, deleting 164 amino acids from the N-terminus. Surprisingly, the missing N-terminal coding segment is present in the frame in the *C. suecicum* genome immediately upstream of the modelled start site. The *C. pallidicaule* splice sites are present as well, so that by using the initiating methionine and splice sites of the *C. pallidicaule* gene, the *C. suecicum* predicted protein would be the same length and align well with the predicted *C. pallidicaule* protein across their entire lengths, with overall 96% identity. This revised gene model was used for the *C. suecicum* putative homolog shown in Figure S12.

In *C. quinoa* there are two genes closely similar to the *C. pallidicaule* gene, on homologous chromosomes from the A and B sub-genomes. These are spliced similarly to the *C. pallidicaule* gene and are highly identical across their entire lengths with each other and the *C. pallidicaule* and revised model *C. suecicum* genes. In *C. berlandieri nuttaliae*, again there are two genes clearly homologous to the *C. pallidicaule* and *C. quinoa* genes, on homologous A and B sub-genome chromosomes as with *C. quinoa*. However, a different splicing pattern is used for both genes, with an upstream in-frame GT donor site, leading to internal deletions of 65 amino acids in both sub-genomic gene models. As with *C. suecicum*, the splicing sites from *C. pallidicaule* or *C. quinoa* are present in both *C. berlandieri nuttaliae* genes. When these are used, the predicted protein products are highly identical to the other genes across their entire lengths. Revised gene models were therefore used for the *C. berlandieri nuttaliae* genes. Again, it is not obvious what the basis is for the different initiation and splice sites as modelled for some of the other *Chenopodium* species genome annotations.

In our *C. berlandieri* assembly, the *C. quinoa* sub-genome A gene has a clear homolog on a single assembled scaffold, which when interpreted with the equivalent *C. quinoa* initiation and splice sites predicts a protein very similar across its entire length to the other *Chenopodium* species genes. Sequences highly similar to the *C. quinoa* sub-genome B homolog are present on multiple scaffolds in our assembly, but a coherent gene model could not be constructed from the fragments; it is not clear whether the sub-genome B gene is rearranged and inactivated, or whether there are sequences missing in the assembly.

Overall, the seven putative *IKU2* homologs in these five genome assemblies are extremely similar, with several sites consistently different between the sub-genome A/*C. pallidicaule* and sub-genome B/*C. suecicum* homologs (Figure S12). There is one non-conservative missense change in the predicted protein from our assembly, a serine at position 470 which is a proline in all six other homologs. These are leucine-rich repeat domain proteins, and the P-to-S change is in one of the leucine-rich repeats, but no other specific biochemical functions are predicted for this sequence.

Although the *A. thaliana MINI3* gene gave multiple TBLASTN hits in the *Chenopodium* genomes, they were all over short protein segments; in none of the genomes was there an extended clearly homologous gene.

#### 2.5.3. *TTG2*

Transparent testa glabra 2 (*TTG2*, also known variously as ATWRKY44, DR. STRANGELOVE 1, DSL1, F3G5.5, F3G5_5, or WRKY44 in the NCBI entry for the *Arabidopsis thaliana* gene NP _181263) is a transcription factor whose mutant phenotypes in *A. thaliana* include altered seed color [35], and reduced seed size [36,37]. A putative ortholog is differentially expressed in relation to seed development in *Fagopyrum esculentum* (common buckwheat) [38]. Thus *TTG2* is a good candidate gene that might vary between domesticated and wild *Chenopodium* plants.

We first identified potential homologs of *Arabidopsis TTG2* in the genomes of *C. pallidicaule* and *C. suecicum*. There were two such genes in *C. suecicum* (Figure S13A), of which one (BBB18587) was more similar to the *Arabidopsis* gene by percent identity (37% vs. 30%). There were six genes in *C. pallidicaule* similar to *A. thaliana TTG2* (Figure S13B), of which one (CP003660) was the most similar to the *Arabidopsis* (and the *C. suecicum*) genes by Clustal Phylogeny (Figure S13C). As annotated in the CoGe database, there is an inconsistency in the spliced mRNA when aligned to the genomic sequence; the first intron donor site does not respect the canonical GT splice site. The protein predicted from this mRNA appears consistent with the *A. thaliana* gene at the N-terminus, hence the protein predicted from this mRNA was used as annotated. RNA-seq was performed for the *C. pallidicaule* genome [39], so conceivably the annotation software employed an atypical isoform of the first intron, or possibly the genomic sequence at this donor site is incorrect. In the case of *C. suecicum*, the mRNA aligned to the genomic sequence respects the canonical splice junctions, but oddly the open reading frame as annotated stops one amino acid before the in-frame stop codon in the genomic sequence, hence the protein as annotated, ending with PAGKEI, is one residue shorter than predicted for translation (Figure S13D). The extra amino acid (R) is added to the version of the gene annotation used here. The *C. pallidicaule* and *C. suecicum* genes are very similar (82% identity overall) with assorted single or short multiple amino acid differences, except for two extended internal insertions/deletions. As in the case of the *SOS1* gene, the extended insertion/deletions in the *C. suecicum* and *C. pallidicaule TTG2* homologs are due to alternative splicing events in the two annotated gene models. In this case, the alternative splice patterns are not interchangeable in the two species; in both cases, modelling the other species’ splice pattern leads to protein truncations (*data not shown*).

Using either of the diploid *TTG2* genes to search the *C. quinoa* and *C. berlandieri nuttaliae* genomes, two closely similar genes were found in each of the tetraploids, on homologous chromosomes. In each case, the sub-genome A homolog was closer to the *C. pallidicaule* gene, and the sub-genome B homolog was closer to the *C. suecicum* gene.

In *C. quinoa*, the sub-genome A homolog (CQ026523) gene model has a GT-to-GC variation at the equivalent first exon splice donor site in both the mRNA and CDS features, which is expected to inactivate that splice site, leading to the absence of the normal protein translation product (Figure S13E). There is no other nearby potential in-frame GT donor site in either the adjacent exon or intron near the inactivated site. Retention of the 76 bp intron does not result in an open reading frame equivalent to the sub-genome B homolog. The gene model for the *C. quinoa* sub-genome B gene (CQ001086) uses canonical splice sites and generates an open reading frame consistent with the diploid species genes. Modelling the splice patterns from either the *C. suecicum* gene or the *C. quinoa* sub-genome B gene (the two are essentially identical splice patterns) with the *C. quinoa* sub-genome A genomic sequence did generate the equivalent spliced isoform, indicating that the alternative splice sites are present in the A sequence, although several small insertions and deletions in the sub-genome A genomic sequence resulted in a frameshift severely truncating the predicted protein (Figure S13F). Thus, it seems unlikely that this *C. quinoa* genome contains a functional sub-genome A *TTG2* homolog.

In *C. berlandieri nuttaliae*, there were two putative homologs based on the closest match to either the quinoa sub-genome B or *C. suecicum* or *C. pallidicaule* gene at the protein level using TBLASTN: Cb002931 annotated on chromosome C6B, and Cb043786 annotated on chromosome C6A. The third closest gene in *C. berlandieri nuttaliae* had a much lower identity than either of the query genes. The *TTG2* homologous sequence on sub-genome A chromosome 6A does not encode an equivalent homologous protein as annotated in the database. The homologous sequence on sub-genome B chromosome 6B encodes a protein similar to the *C. pallidicaule TTG2* protein but is missing approximately 50 amino acids of the C-terminus corresponding to the last two coding exons. Modelling the splice patterns from the *C. quinoa* sub-genome B gene on the *C. berlandieri nuttaliae* sub-genome A genomic sequence generates an equivalent spliced isoform to the *C. quinoa* gene, but as with the *C. quinoa* sub-genome A genomic sequence, the *C. berlandieri nuttaliae* sub-genome A spliced isoform has insertions/deletions which cause a frameshift, similarly eliminating the correct open reading frame (data not shown, but essentially the same as in Figure S13F). Thus, as with *C. quinoa*, this *C. berlandieri nuttaliae* genome may lack a sub-genome A *TTG2* homolog. Modelling the splice pattern from the *C. quinoa* sub-genome B gene on the *C. berlandieri nuttaliae* sub-genome B genomic sequence as defined by the database generates the same open reading frame as is already annotated in the database for that gene, lacking the last two coding exons. When the *C. quinoa* sub-genome B mRNA is aligned to a longer segment of the homologous chromosomal region of *C. berlandieri nuttaliae* chromosome 6B (manually including sequences downstream from the annotated 3′ end of the gene in the database), the last two coding exons are indeed present in the genomic sequence and can be spliced per the *C. quinoa* gene model, and an open reading frame is generated equivalent to the full quinoa sub-genome B and diploid genome gene versions (Figure S13G).

In our *C. berlandieri* assembly, there were only two scaffolds with extended homology to the *C. quinoa* CQ001086-encoded protein using TBLASTN. Using the CQ001086 mRNA to model the splice pattern, both scaffolds contained all the exons and could be spliced to create the same patterns as in *C. quinoa* and *C. berlandieri nuttaliae.* One scaffold in our assembly, scaffold4052, when spliced, had the same insertion/deletions as in the other two tetraploids, leading to the same frameshift and truncated protein. The other scaffold in our assembly, scaffold27337, generated a full-length protein similar to the other two tetraploid B sub-genome copies when spliced. Thus, all three of these tetraploid genomes appear to lack a function *TTG2* homolog in their A sub-genomes. It should be noted that a shorter version of the *TTG2* protein can be generated from initiation at an internal methionine downstream of the A sub-genome indels causing the frameshift. There is no way to know from these data whether translation initiation from an ATG so far from the 5′ end of the presumed spliced mRNA is possible or is occurring.

Overall, the *TTG2* homologs are similar, especially the tetraploid B sub-genome copies and *C. suecicum* (Figure S13H). The *C. quinoa* gene has a substitution at position 349 of N for H present in the other B sub-genome genes, but the *C. pallidicaule* gene also has an N at the equivalent position. The gene in our assembly has a T at position 406, which is an I in all the other genes, a non-conservative change of a hydrophobic residue for a hydrophilic residue that could potentially be phosphorylated. As with the *A. thaliana TTG2* gene, the predicted protein product of the spliced de novo assembly B sub-genome gene contains two WRKY domains, a conserved feature of a large family of plant transcription factors. The amino acid substitution at position 406 in our assembly is slightly proximal to the second WRKY domain (Figure S13I), but could potentially modulate the activity of that domain. Even more proximal, beginning at position 381 is a short run of basic residues that is proposed to function as a nuclear localization signal, that is intact in all the complete coding gene models [40].

## 3. Discussion

### 3.1. Discrimination of Chenopodium Species

The various members of the *Chenopodium* species complex are notoriously challenging to identify in the field [41]. A variety of barcode markers have been suggested for discriminating all members of the genus with DNA sequencing [15,17,18,42,43]. In some practical situations, however, a universal marker is not required; rather, what is needed is a robust assay for distinguishing the species likely to be encountered in a specific region. In eastern North America, among the most problematic species to distinguish are *C. berlandieri* and *C. album*. These are practically indistinguishable by general plant or leaf morphology, although they may be resolved by examination of mature seeds under sufficient magnification (R. Jellen, pers. comm.) Seed characters are not present early in the growing season, however. Our long-term interest is in the genetic diversity of *C. berlandieri* in Canada, to be assessed through sequencing of plants collected across the country. For that purpose, it is important to be able to assess the species identity of younger plants collected at earlier times in the growing season (typically May/June through September/October in southern Canada).

The barcode markers we have employed appear well-suited for distinguishing *C. berlandieri* from *C. album*. The specific amplicons of *rbcL* and *matK* that we analyzed contain a total of seven clearly resolved sequence differences among the various plants we have tested. In all cases, all seven of the variant sites are found together, as either the *C. berlandieri* or *C. album* haplotype. These markers would of course not be completely useful in distinguishing *C. berlandieri* x *C. album* hybrids. In general, fertile hybrids between these two species should be unlikely due to their different ploidy (tetraploid versus hexaploid, presumably leading to pentaploid sterile hybrids) [44]. We also note in passing that these two barcoding genes can also distinguish *C. berlandieri* from other Canadian *Chenopodium* species (details to be published elsewhere).

*C. berlandieri* and *C. quinoa* are almost identical in the chloroplast gene amplicons we have tested, thus our barcode markers would not robustly distinguish between these two species. At present, there is no realistic scenario where these species might be confused in Canadian field collections, although the young plants of both are also quite similar morphologically. So far at least, quinoa is only grown in Canada in well-defined commercial or experimental fields. Conceivably, additional work might be needed in the future to distinguish *C. berlandieri* from *C. quinoa*, if very large acreages of quinoa are cultivated.

### 3.2. Genome Sequencing and Assembly of a Canadian C. berlandieri Accession

To our knowledge, this is the first reported assembly and annotation of *Chenopodium berlandieri.* An assembly exists for another accession of *C. berlandieri* from Mexico, subspecies *nuttaliae* [13], which has not been annotated in the literature yet. It is unclear how domesticated the Mexican accession is, but it is potentially highly so. Our assembly appears almost as much diverged from this Mexican accession as from the published genome of *C. quinoa*, thus regardless of the exact nature of the Mexican material, our sequenced accession is very different genomically, and may be a better representation of wild North American or at least Canadian *C. berlandieri*. Genomes of five additional accessions of *C. berlandieri* are reported in the KAUST *Chenopodium* database (https://www.cbrc.kaust.edu.sa/chenopodiumdb/ (accessed on 6 January 2023)), presumably wild, but those genomes are not assembled or annotated. Our assembly, therefore, adds significant new information to the small number of assembled *Chenopodium* genomes.

In general, our assembly shows many expected properties. The total assembled genome size is slightly smaller than, but is consistent with those of the *C. quinoa* and *C. berlandieri nuttaliae* tetraploid genomes (approximately 1.4 Gb). The repetitive sequence content and distribution are very similar to that reported for *C. quinoa*. Almost 90% of BUSCO-defined eudicot genes could be found in the assembly. *C. berlandieri* is reported primarily to self-fertilize [D. Brenner, D. Jarvis, pers. comm.], which is consistent with the paucity of heterozygous SNPs in non-repetitive sequences (1 per 3000–4000 bp).

Our assembly does appear to have resolved the two parental sub-genomes A and B correctly, at least for protein-coding genes. In all the specific genes we examined, each of the two quinoa sub-genome homologs could be aligned to one or the other scaffold of our assembly, with little ambiguity. For genes that are fragmented into multiple scaffolds in our assembly, we were able to generate complete models that are likely to combine correctly scaffolds from the two parental sub-genomes. Nonetheless, gene annotation would be significantly improved or at least made easier with the addition of long reads of genomic DNA and RNA-Seq data. We hope to obtain such data in the future.

In comparing our genome to those of *C. quinoa* (version 2 in the CoGe database) and *C. berlandieri nuttaliae* (in the CoGe database), it was unexpected that our assembly had almost equivalently large numbers of differences from the other two assemblies even in non-repetitive regions. Nonetheless, there were slightly fewer differences between our sequenced genome and that of *C. berlandieri nuttaliae*, and more with that of *C. quinoa*, consistent with the two *C. berlandieri* genomes being more closely related to each other than to the *C. quinoa* genome.

On a technical point, it seems anomalous that there should be more heterozygous SNPs identified by aligning our sequence reads against the *C. quinoa* and *C. berlandieri nuttaliae* genomes than were called against by aligning the reads to our own assembly, since calling heterozygous SNPs implies that there were individual reads carrying one or the other of both alleles. If so, then those sites should also be called as heterozygous in our assembly. Most likely, many of these are false positive calls generated by the alignment of the raw sequence reads to the other genomes. The Platanus assembly algorithm and the pipeline of bwa_mem, mpileup, and bcftools call, undoubtedly have different stringency parameters, which would be very difficult if not impossible to make fully consistent due to the way the parameters are internally defined. On visual inspection, using IGV, of heterozygous sites called in the other genomes, many of the calls are adjacent to repeat masked sequences, and may represent mis-alignments of repetitive sequences that were not masked.

### 3.3. Analysis of Genes Involved in Agricultural Traits

Comparison of genes known to play roles in agriculturally important traits and domestication, among the various wild and domesticated *Chenopodium* species genomes, should provide useful information toward understanding their different physiologies. We analysed several such genes of known relevance, with the immediate goal to justify the general approach, not doing a comprehensive analysis of all such genes. We generated gene models from our assembly by comparison with the assembled and annotated genomes of the tetraploids *C. quinoa* (v2), *C. berlandieri nuttaliae*, and the diploids *C. pallidicaule* (closer to parental sub-genome A) and diploid *C. suecicum* (closer to parental sub-genome B).

#### 3.3.1. *SOS1*

In the most intriguing comparison, our Canadian *C. berlandieri* genome appears to lack one of the expected two *SOS1* (salt overly sensitive 1) homologs, the sub-genome A copy. *SOS1* encodes a sodium ion transporter, and regulation of *SOS1* is of considerable interest for groups attempting to adapt quinoa (and other plants) to cultivation in high-salinity soils (such as in the Middle East) [4,29,30,45]. As noted above, in parts of Canada, excessive rainfall rather than high soil salinity is a greater problem for quinoa adaptation. We speculate that the loss of one copy of *SOS1* might be of selective advantage for a *Chenopodium* growing in wet areas such as southern Manitoba. If so, then deleting one copy of *SOS1* might conceivably improve the adaptation of quinoa to areas that are too wet for good adaptation currently. It remains to be seen whether the inactivation of one *SOS1* homolog is found in other *C. berlandieri* accessions in Canada, or even in accessions of quinoa adapted to wetter climates such as coastal South America or the U.S. Pacific northwest.

#### 3.3.2. *TTG2*

A second observation meriting further investigation is the apparent lack of a functional A sub-genome homolog of *TTG2* in all three tetraploid genomes analyzed here. *TTG2* plays a key role in seed coat color, through the regulation of genes involved in the intracellular transport of proanthocyanidins (tannins) [35,46]. *TTG2* is also involved in seed size, as noted for *Arabidopsis* mutations and in a network study of agronomic traits in barley [36,37,47]. Conceivably, selection for a gene knockout might have occurred in quinoa selected for lighter seed color, but that would not be consistent with the typical black seed color of our Canadian *C. berlandieri* accession. The color of the *C. berlandieri nuttaliae* seeds is not known to us. Similarly, a shared loss of gene dosage of *TTG2* is not consistent with the difference in seed size between domesticated *C. quinoa* and *C. berlandieri nuttaliae* and our wild *C. berlandieri*. Further confirmation of the non-functional A homolog in all these plants would be desirable.

Otherwise, there are single amino acid non-conservative differences in our assembled genome, in *TTG2* and *IKU2* compared to the other *Chenopodium* species. These are candidates for interesting variants but await further confirmation by sequencing additional examples of all these species.

#### 3.3.3. Utility of Genomic Comparisons

As a general point, the differences in gene sequences that we observed among the *Chenopodium* genomes should be further explored in multiple accessions of each species, to distinguish between intra- and inter-species variation.

The comparison of gene annotations had unexpected challenges. For many of the genes we examined, the spliced coding sequence gene models annotated in the CoGe database are partly inconsistent with each other or with the genomic sequence of the same genomes, particularly in terms of the inclusion or exclusion of alternatively spliced exons. It is not clear why the homologous genes are differently annotated in these cases. Nonetheless, our preliminary analyses support the utility of studies of genomic variation among these *Chenopodium* species, for genes known to be involved in modulating agriculturally important traits.

## 4. Materials and Methods

### 4.1. Plant Collection and Germination

*Chenopodium berlandieri* plants were previously collected at various sites along the Red River in southern Manitoba [8,19]. Leaf and seed material was stored dry. Seeds were also stored from daughter generations of plants grown under controlled field conditions, starting from the initially collected wild plant seeds. For barcoding and whole genome sequencing, plants were newly grown from second-generation seeds from the SV site [8,19]. Commercial quinoa seeds were purchased from West Coast Seeds, Inc. (Vancouver, BC, Canada) (variety RedHead), germinated, and grown for fresh leaf material in the greenhouse of the Université de Montréal Institut de Recherche en Biologie Végétale (IRBV). Seeds were graciously provided from the USDA North Central Regional Plant Introduction Station at Iowa State University for *C. berlandieri var zschackei* (accession Ames 33021) and *C. album* (accessions Ames 29960, 32979, and 33032), and similarly germinated and grown in the IRBV greenhouse.

### 4.2. DNA Purification

DNA was prepared from leaf material using a variant of the CTAB protocol of OPS Diagnostics (https://opsdiagnostics.com/ (accessed on 11 November 2022)), with the details to be published elsewhere. DNA was assayed by electrophoresis on 1% Tris-acetate agarose gels with SafeRed fluor (Applied Biological Materials) and photographed using an ImageQuant (Amersham). DNA was considered good quality if a diffuse band was readily observed at approximately 20 kb (the resolution limit of the gel matrix).

### 4.3. Primer Design, PCR, and Sanger Sequencing

Primers for amplification and sequencing of *rbcL* and *matK* were designed using NCBI Primer-BLAST (https://www.ncbi.nlm.nih.gov/tools/primer-blast/ (accessed on 13 August 2021)), and confirmed with Primer3Plus (https://www.bioinformatics.nl/cgi-bin/primer3plus/primer3plus.cgi (accessed on 13 August 2021)) (Table 5).

PCR was performed using Phusion DNA polymerase (Fisher Scientific, Ottawa, ON, Canada), with the standard HF buffer, using a hot start protocol; full details are in the Supplemental Materials. PCR products were sequenced at the Centre d’Expertise et Services of Genome Quebec, situated at CHU Ste-Justine in Montreal, Quebec, Canada, using the same PCR primers. In most cases, both directions were sequenced, and were always consistent, although only one direction is shown in some of the figures.

### 4.4. Genomic Sequencing

Sequencing was performed at the Centre d’Expertise et Services of Genome Quebec. For details see the Supplemental Materials.

### 4.5. Informatics

#### 4.5.1. PCR-Sanger Sequencing

Sanger sequence traces from the Genome Quebec core laboratory were analyzed with CodonCode Aligner. Reference sequences were obtained from the NCBI Gene and Nucleotide databases.

#### 4.5.2. Whole Genome Sequencing and Assembly

Whole genome sequence assembly and analysis were performed on the Compute Canada system, now the Digital Research Alliance of Canada, using FastQC [48], KMC3 [49], GenomeScope [50], and FastP [51]. Genome assembly was performed using both Platanus [52] and Abyss [53]. Statistics of the assemblies were compared using scripts in the BBTools suite (https://sourceforge.net/projects/bbmap (accessed on 19 March 2022), or with QUAST [54]. Genome completion was assessed using BUSCO (Benchmarking Universal Single-Copy Orthologue) [55] to search our assemblies for homologs of a set of annotated genes of eudicot plants. Repeat sequence content was assessed using RepeatMasker [27,56], with the quinoa repeat library in PlantRep [27]. For additional details, see the Supplemental Materials.

Platanus assembly involves three stages and generates three successive data sets, named contigs, scaffolds, and gap-closed scaffolds. We report the results after the third stage, although there are only minor differences between the second and third stages. In contrast, the assembly was significantly condensed going from first-stage contigs to second-stage scaffolds. Since only a single insert library was used, these scaffolds result from paired-end read information incorporated for individual inserts longer than 300 bp (twice the read length). Typically, in genome assemblies, scaffolds are considerably larger than primary contigs; in our case, they are only moderately larger, but we use the Platanus terminology of ‘scaffolds’ for consistency with the software.

The genome assembly is published on the CoGe website of CyVerse (https://genomevolution.org/coge/OrganismView.pl (accessed on 17 October 2022)) under the genus *Chenopodium* (id44708, not to be confused with the completely different assembly of *Chenopodium berlandieri subsp. nuttaliae*). A maximum of 200,000 contigs/scaffolds are allowed by the system, thus the assembly includes all scaffolds larger than 500 bp and some but not all scaffolds of 500 bp. These short scaffolds probably contain mostly repetitive sequences. Thus it is expected that most unique sequences including gene coding sequences are included in the database. Raw sequence reads of the forward and reverse FastQ files are published in the NCBI Sequence Read Archive (SRA), accession number SRR22093186, associated with BioProject accession number PRJNA895488.

#### 4.5.3. Variant Calling

Variant calling was performed with sambamba [57], bwa [58], and bcftools (specifically mpileup and call) [59,60]. Variants in non-repetitive sequence were filtered using BEDOPs [61] and BEDTools [62]. For variant calling, the genomes of *C. quinoa* (version 2) and *C. berlandieri subsp. nuttaliae* were downloaded in FastA format from the CoGe database (https://genomevolution.org/coge/ (accessed on 15 June 2022)). For additional details see the Supplemental Materials.

#### 4.5.4. Gene Models and Comparisons between Chenopodium Species

For sequence comparison of individual genes (e.g., *SOS1*), reference sequences and spliced coding region gene models were obtained for the genes in question from the *C. pallidicaule*, *C. suecicum*, *C. quinoa* version 2, and *C. berlandieri subsp.* The *nuttaliae* (in the text the name is simplified to just *C. berlandieri nuttaliae*) annotated genomes in the CoGe database (https://genomevolution.org/coge/ (accessed on 30 January 2022)). As described in Results, gene models found in CoGe were revised for maximum consistency of coding exon inclusion, based on the comparison of the various species. Potential orthologs (referred to simply as homologs) were identified in our *C. berlandieri* genome assembly, and gene models were constructed. Splign, SIM4, and GMAP were all used for model building, with essentially identical results [63,64,65]. Splign provides more easily interpreted graphics, while GMAP produces text output that is easier to manually assemble into full-length spliced mRNA gene models. Re-annotated genes were aligned using Clustal Omega and graphics were generated using Jalview.

#### 4.5.5. Visualization of Sequence Alignments and Graphic Outputs

Genomic alignments and called variants were visualized with the Integrative Genomics Viewer (IGV) [66]. Pairwise dot plots were generated with the online server of D-GENIES [67]. Quinoa reference organelle genomes were chloroplast NC_034949 (152,099 bp) [22], and mitochondrion NC_041093 (315,003 bp) [68].

## 5. Conclusions

We optimized barcode markers for discriminating between *C. berlandieri* and *C. album* in field collections that might be made before the appearance of mature seeds, late in the growing season. We then sequenced and assembled an accession of Canadian *C. berlandieri*, obtained from Manitoba. The assembly was near the expected size of 1.4 Gb, and had the properties expected for an allotetraploid containing the *Chenopodium* A and B sub-genomes. Although the assembly was fragmented, we were able to call variant sites in the genome, and compare the structures of several agriculturally important genes among multiple *Chenopodium* species. We identified an intriguing difference between *C. quinoa* and the Canadian *C. berlandieri* in the *SOS1* gene, one copy of which appears to be non-functional in Canadian but present in Mexican *C. berlandieri* and in *C. quinoa*. This difference may be relevant to the adaptation of the Canadian accession to local wet growing conditions. Similar promising observations have been made through the sequencing of wild *C. ficifolium*, and comparisons of the flowering locus genes [9]. Our results support the utility of sequencing and assembling genomes of additional wild *Chenopodium* species.

## Figures and Tables

**Table 1:** GenomeScope model kmer = 50, forward reads trimmed to remove adapter and poly-G sequences.

Property	Min	Max
Heterozygosity	0.0590213%	0.0666777%
Genome Haploid Length	1,041,758,722 bp	1,044,841,788 bp
Genome Repeat Length	204,619,403 bp	205,224,971 bp
Genome Unique Length	837,139,319 bp	839,616,817 bp
Model Fit	96.9115%	99.3631%
Read Error Rate	0.0154969%	0.0154969%

**Table 2:** Assembly statistics for Platanus after the three steps of contig assembly, scaffolding, and gap-closing. Parameters: k-mer 96, step size default, bubble crush default (0.1), min coverage default (2). Main genome scaffold N/L50: 42,216/6.641 KB. Main genome contig: N/L50 54,953/5.258 KB. Main genome scaffold: N/L90 1,432,405/105. Main genome contig: N/L90 1,464,507/105. Max scaffold length: 126.758 KB. Max contig length: 79.472 KB. Number of scaffolds > 50 KB: 304. Percent main genome in scaffolds > 50 KB: 1.46%. GC content: 0.37.

Minimum Scaffold Length	Number of Scaffolds	Number of Contigs	Total Scaffold Length	Total Contig Length	Scaffold Contig Coverage
All	2,574,694	2,606,797	1,242,701,758	1,241,539,883	99.91%
50 bp	2,574,694	2,606,797	1,242,701,758	1,241,539,883	99.91%
100 bp	2,574,694	2,606,797	1,242,701,758	1,241,539,883	99.91%
250 bp	318,491	350,594	991,976,458	990,814,583	99.88%
500 bp	225,542	257,463	958,701,073	957,548,972	99.88%
1 KB	150,725	181,935	905,768,428	904,653,031	99.88%
2.5 KB	86,640	115,773	805,392,537	804,378,182	99.87%
5 KB	53,877	79,681	688,614,217	687,732,632	99.87%
10 KB	26,945	46,328	496,895,973	496,241,228	99.87%
25 KB	4592	11,168	156,852,731	156,634,629	99.86%
50 KB	304	1088	18,154,363	18,128,771	99.86%
100 KB	2	11	229,032	228,698	99.85%

**Table 3:** Repetitive sequence content of Platanus k96 assembly, assessed by RepeatMasker version 4.1.1, run with rmblastn version 2.10.0+, query was compared to classified sequences in “quinoa_repts.fa”.

Type of Element	Number of Elements in Class	Total Sequence Length of Elements in Class	Percentage of Genome in Class
**Retroelements**	1,633,307	451,748,765 bp	36.35%
			
**SINEs**:	3097	322,078 bp	0.03%
Penelope	379	162,757 bp	0.01%
			
**LINEs**:	50,776	19,027,981 bp	1.53%
CRE/SLACS	2499	879,877 bp	0.07%
L2/CR1/Rex	6935	628,484 bp	0.05%
R1/LOA/Jockey	2472	710,340 bp	0.06%
R2/R4/NeSL	5246	1,337,187 bp	0.11%
RTE/Bov-B	6705	1,395,665 bp	0.11%
L1/CIN4	23,615	13,263,676 bp	1.07%
			
**LTR elements:**	579,434	432,398,706 bp	34.80%
BEL/Pao	0	0 bp	0.00%
Ty1/Copia	352,751	94,725,050 bp	7.62%
Gypsy/DIRS1	1,218,402	336,023,692 bp	27.04%
Retroviral	2547	964,904 bp	0.08%
			
**DNA transposons**	299,635	60,725,696 bp	4.89%
hobo-Activator	76,873	15,027,731 bp	1.21%
Tc1-IS630-Pogo	46,962	8,490,986 bp	0.68%
En-Spm	0	0 bp	0.00%
MuDR-IS905	0	0 bp	0.00%
PiggyBac	0	0 bp	0.00%
Tourist/Harbinger	12,861	87,074 bp	0.21%
Other (Mirage, P-element, Transib)	0	0 bp	0.00%
			
**Rolling-circles**	1407	327,768 bp	0.03%
**Unclassified:**	1,299,035	248,765,564 bp	20.02%
**Total interspersed repeats:**		761,240,025 bp	61.26%
**Small RNA:**	3827	561,483 bp	0.05%
**Satellites:**	12,272	2,115,469 bp	0.17%
**Simple repeats:**	316,588	14,181,697 bp	1.14%
**Low complexity:**	58,004	3,046,351 bp	0.25%

**Table 4:** BUSCO analysis of Platanus k96 assembly using a database of eudicot-specific genes. BUSCO v. 5.2.2. The lineage dataset is: eudicots_odb10 (Creation date: 10 September 2020, number of genomes: 31, number of BUSCOs: 2326). BUSCO was run in mode: genome. Gene predictor used: metaeuk.

Number of Genes	Gene Status
1859	Complete BUSCOs (C)
645	Complete and single-copy BUSCOs (S)
1214	Complete and duplicated BUSCOs (D)
181	Fragmented BUSCOs (F)
286	Missing BUSCOs (M)
2326	Total BUSCO groups searched

**Table 5:** Primer sequences. Forward and reverse orientation is defined with forward being the coding strand of the genes, all of which encode proteins. Melting temperatures are per NCBI Primer-BLAST.

Primer	Gene	Strand	Sequence (5′-3′)	Tm (°C)
rbcLM1-F	rbcL	Forward	CGGTATCCAAGTTGAGAGAG	55
rbcLM1-R	rbcL	Reverse	TAAATACCACGACTTCGGTC	55
matK390-F	matK	Forward	CGATCTATTCATTCAATATTTC	49
matK3FKIM-R	matK	Reverse	CGTACAGTACTTTTGTGTTTACGAG	58

## Data Availability

Raw sequence reads of the forward and reverse FastQ files are published in the NCBI Sequence Read Archive (SRA), accession number SRR22093186, associated with BioProject accession number PRJNA895488. The genome assembly is published on the CoGe website of CyVerse (https://genomevolution.org/coge/OrganismView.pl (accessed on 17 October 2022)) under the genus Chenopodium (id44708).

## Supplementary Materials

The following supporting information can be downloaded at: https://www.mdpi.com/article/10.3390/plants12030467/s1. Table S1. Statistics of Abyss genome assembly with k-mers = 104. Table S2. BUSCO analysis of Abyss k104 assembly using a database of eudicot-specific gene. Figure S1. Primer design of rbcL. Figure S2. *rbcL* barcoding. Figure S3. Primer design of matK. Figure S4. *matK* barcoding. Figure S5. Selected scaffolds showing examples of heterozygous SNPs. Figure S6. Chloroplast genome assembly. Figure S7. Mitochondrial genome assembly. Figure S8. Alignment of *C. quinoa* chloroplast and mitochondrial genomes to each other. Figure S9. Gene models of *SOS1* sub-genome B. Figure S10. Gene models of *SOS1* sub-genome A. Figure S11. *IKU1* multiple sequence alignment. Figure S12. *IKU2* gene models. Figure S13. *TTG2* gene models.
