# Supplementary material for: Genomic Sequence of Canadian Chenopodium berlandieri: A North American Wild Relative of Quinoa
Source: Plants (Basel). 2023 Jan 19;12(3):467. doi: 10.3390/plants12030467 (PMC9920564; doi:10.3390/plants12030467)
Supplement: Supplementary file 1 [file plants-12-00467-s001.zip › plants-2109851-supplementary.pdf]

Supplemental Materials

1. Materials and Methods

1.1 Primer design

An advantage to the NCBI Primer-BLAST is that it can accept input of multiple sequences in a single fasta file, and if possible, generate primers usable with all the input sequences.

1.2 PCR protocol

PCR was performed using Phusion DNA polymerase (Fisher Scientific), with the standard HF buffer, using a hot start protocol: a 30 second precycle at 98°C, followed by 40 cycles (30 seconds melting at 98°C, 30 seconds annealing at the defined temperature, 30 seconds extension at 72°C), followed by 10 minutes at 72°C then stopping at 4°C. For all the primers employed in this study, annealing was successful at either 57°C. PCR was assayed on 1% Tris-acetate agarose gels with SafeRed to visualize DNA.

1.3 Genome sequencing

Sequencing was performed at the Centre d'Expertise et Services of Genome Quebec. 2x151bp paired-end sequencing was done on an Illumina NovaSeq 6000 instrument. 320,869,080 total paired reads were generated, totalling 96.9 Gbp of raw sequence, or 69-fold coverage assuming a genome size of 1.4 Gbp. Per FastP, the average insert size of the input library was estimated to be 218bp, hence there was overlap between the forward and reverse read fastq files for many inserts.

1.4 Informatics

1.4.1 Sanger sequencing

Sanger sequence traces from the Genome Quebec core laboratory were analyzed with CodonCode Aligner (https://www.codoncode.com/aligner/). After loading the traces, bases were called and sequences were trimmed using the suite tools, followed by contig assembly. As needed, sequences of the relevant genes from the relevant species were directly loaded as fasta files, and included in the contig alignments. Sequences of genes from various *Chenopodium* species were obtained from the NCBI Nucleotide database, or from the iBOLD database (https://www.boldsystems.org/) maintained at the University of Guelph in Ontario, Canada.

1.4.2 Raw sequence quality control, cleanup, and genome size estimate by k-mer analysis

For analysis of the whole genome sequences, raw data quality was assessed using FastQC [1]. K-mer analysis was performed using KMC3 [2]. We compared this to Jellyfish, and the two tools generated similar results but KMC3 was significantly easier to implement, with the option to run it locally on a Windows OS system. KMC3 output was modelled using the online GenomeScope server [3]; models could be generated using k25 and k50 counts, with similar results, but GenomeScope was unable to generate a model using a k100 count.

The raw Illumina fastq files were cleaned using FastP [4], with parameters restricted to removing adapter sequences and polyG sequences, the latter being a known systematic issue with NovaSeq chemistry. The documentation for Platanus recommends not performing pre-assembly base quality

trimming, as quality trims are intrinsic to the Platanus assembler, which works best with the most complete input sequence files especially with paired end data files.

1.4.3 Whole genome assembly

Genome assembly was performed using both Platanus [5] and Abyss [6], trying a variety of parameters including different initial k-mers and allowed mismatch percentages. We used Platanus v1. We did not attempt to use Platanus v2 (allee) as we expected minimal heterozygosity and it is unclear how Platanus-allee deals with polyploid genomes versus haplotypes or diploids. Statistics of the assemblies were compared using scripts in the BBTools suite (https://sourceforge.net/projects/bbmap), and with QUAST [7]. Note that the BBTools output reverses the N50/L50 statistics with respect to standard nomenclature.

1.4.4 Repeat sequence analysis

Repeat sequences were assessed using RepeatMasker version 4.1.1 [8], run with rmblastn version 2.10.0+; query was compared to a set of quinoa genome repeat elements annotated in the PlantRep database [9].

1.4.5 BUSCO analysis of standardized gene set completion

Genome completion was assessed using BUSCO (Benchmarking Universal Single-Copy Orthologue) v5.2.2 [10], using the eudicots_odb10 database, based on 31 genomes, as a reference.

1.4.6 Variant calling

Variant calling was performed by aligning the raw fastq reads to the Platanus k96 genome assembly previously generated from those reads. The goal was to assess the degree of heterozygosity, not to call variants with respect to other published genomes. First, sambamba was used to remove duplicate reads, sort and index the raw fastq files [11]. Second, bwa-mem was run with mismatch parameter 6 (default is 4) [12], followed by Bcftools-mpileup with a maximum read depth of 400 to reduce running time (although oddly in the resulting variant vcf files there are still a small number of variants with greater than 400 read depth). Bcftools call was run to call variants, using default stringency parameters [13,14]. For statistics of variant calling the genome assembly, insertion/deletion variants were excluded, and contigs shorter than 300bp were excluded (these are mostly repetitive sequences in any case). To further restrict variants to non-repetitive sequences, BEDOPs was used to convert the RepeatMasker output file to bed format [15]. BEDTools was used to filter the variant vcf file to exclude sites in the RepeatMasker bed file [16]. For variant calling, the genomes of *C. quinoa* (version 2) and *C. berlandieri nuttaliae* were downloaded in fasta format from the CoGe database. We used vcflib stats to obtain variant genotype statistics; bcftools stats gave slightly different numbers for total, heterozygous and homozygous SNPs for unclear reasons, but the differences do not affect our conclusions.

1.4.7 Dot plot comparisons of organelle genomes

Dot plots of genome assembly scaffolds to C. quinoa chloroplast and mitochondrial genomes were performed using the online D-GENIES server [17].

1.4.8 Individual gene model building, intra- and inter-species comparisons

For sequence comparison of individual genes (*e.g. SOS1*), reference sequences and spliced gene models were obtained for the genes in question from the *C. pallidicaule*, *C. suecicum*, *C. quinoa* v2 and *C. berlandieri nuttaliae* annotated genomes in the Comparative Genomics (CoGe) database (https://genomevolution.org/coge/) In some cases, gene models were revised using the spliced model from another species with the genomic sequence of the given species. This improved consistency of gene comparisons across the species, while making the description of the various genes in the Results section somewhat hard to describe. Sources for finding the genes varied, sometimes *Arabidopsis thaliana*, sometimes *C. quinoa* itself. One or more of these reference genes were used to blast our assembled SV89-10 Canadian *C. berlandieri* genome, at either the DNA or protein level, using the Platanus k-mer 96 gap_closed assembly. In some cases, genes were fragmented across multiple scaffolds in our assembly. In such cases, scaffolds containing equivalent coding exons from 5' to 3' were manually combined. Given the two parental sub-genomes of *C. berlandieri*, it was important not to mis-assemble scaffolds containing different exons from each sub-genome; fortunately, in all cases that we examined, individual assembled scaffolds containing protein-coding exons were more similar to either the A or B sub-genome of the reference genomes. In all genes examined, there were no discrepancies between assignment of the A and B homologs between the two tetraploid references (*C. quinoa* v2, *C. berlandieri nuttaliae*). Spliced gene models were built using either the A or B sub-genome homolog from the *C. berlandieri nuttaliae* annotated genes with the manually assembled A or B sub-genome scaffolds of our assembly; if the *C. berlandieri nuttaliae* gene modelling was ambiguous, some of the other reference species were used alternatively. Splign, SIM4 and GMAP were all used for model building, with essentially identical results [18-20].

Minimum scaffold length	Number of scaffolds	Number of contigs	Total scaffold length	Total contig length	Scaffold contig coverage
All	2,223,105	2,228,384	1,308,412,757	1,308,135,794	99.98%
100	2,223,105	2,228,384	1,308,412,757	1,308,135,794	99.98%
250	790,154	795,430	1,091,061,749	1,090,800,272	99.98%
500	351,479	356,753	941,537,391	941,276,024	99.97%
1 KB	176,632	181,906	821,399,569	821,138,202	99.97%
2.5 KB	105,493	110,633	701,949,973	701,694,206	99.96%
5 KB	54,911	59,024	521,340,195	521,133,889	99.96%
10 KB	17,506	19,614	259,613,574	259,507,514	99.96%
25 KB	843	1,074	25,535,499	25,524,026	99.96%
50 KB	9	19	488,766	488,248	99.89%

Table S1. Statistics of Abyss genome assembly with k-mers=104. Main genome scaffold N/L50: 88,430/3.125 KB. Main genome contig N/L50: 91,956/3.02 KB. Main genome scaffold N/L90: 1,218,602/207bp. Main genome contig N/L90: 1,208,431/207bp. Max scaffold length: 76.235 KB. Max contig length: 66.776 KB. Number of scaffolds > 50 KB: 9. Percent main genome in scaffolds > 50 KB: 0.04%. GC content: 0.37.

Number of genes	Gene status in genome assembly
1486	Complete BUSCOs (C)
737	Complete and single-copy BUSCOs (S)
749	Complete and duplicated BUSCOs (D)
426	Fragmented BUSCOs (F)
414	Missing BUSCOs (M)
2326	Total BUSCO groups searched

Table S2. BUSCO analysis of Abyss k104 assembly using a database of eudicot-specific genes. # BUSCO version is: 5.2.2. # The lineage dataset is: eudicots_odb10 (Creation date: 2020-09-10, number of genomes: 31, number of BUSCOs: 2326). # Gene predictor used: metaeuk

Figures

```
NC_034949.1_Cqu   601 AAAGATGATGAAAACGTGAACTCCCAGCCGTTTATGCGTTGGAGAGACCGTTTCCTATTT
NC_034950.1_Cal   601 AAAGATGATGAAAACGTGAACTCCCAGCCGTTTATGCGTTGGAGAGACCGTTTCCTATTT

NC_034949.1_Cqu   661 TGTGCCGAAGCTATTTATAAAGCACAAGCCGAAACAGGCGAAATCAAAGGTCATTACTTG
NC_034950.1_Cal   661 TGTGCCGAAGCTATTTATAAAGCACAAGCCGAAACAGGCGAAATCAAAGGTCATTACTTG

NC_034949.1_Cqu   721 AATGCTACTGCCGCTACATGTGAAGAAATGATAAAAAGGGCTGTATTTGCCAGAGAATTG
NC_034950.1_Cal   721 AATGCTACTGCGGCTACATGTGAAGAAATGATAAAAAGGGCTGTATTTGCCAGAGAATTG

NC_034949.1_Cqu   781 GGAGCTCCTATCGTAATGCATGACTACTTAACAGGGGGATTCACTGCAAATACTAGCTTG
NC_034950.1_Cal   781 GGAGCTCCTATCGTAATGCATGACTACTTAACAGGGGGATTCACTGCAAATACTAGCTTG

NC_034949.1_Cqu   841 GCTCATTATTGCCGAGATAATGGTCTACTTCTTCACATCCACCGTGCAATGCACGCAGTT
NC_034950.1_Cal   841 GCTCATTATTGCCGAGATAATGGTCTACTTCTTCACATCCACCGTGCAATGCACGCAGTT

NC_034949.1_Cqu   901 ATTGATAGACAGAAGAATCATGGTATGCACTTCCGTGTACTAGCTAAAGCATTACGTCTG
NC_034950.1_Cal   901 ATTGATAGACAGAAGAATCATGGTATGCACTTCCGTGTACTAGCGAAAGCATTACGTCTG

NC_034949.1_Cqu   961 TCTGGTGGAGATCATATTCATTCTGGTACCGTAGTAGGTAAGCTTGAAGGAGAAAGAGAT
NC_034950.1_Cal   961 TCTGGTGGAGATCATATTCATTCTGGTACCGTAGTAGGTAAGCTTGAAGGAGAAAGAGAT
```

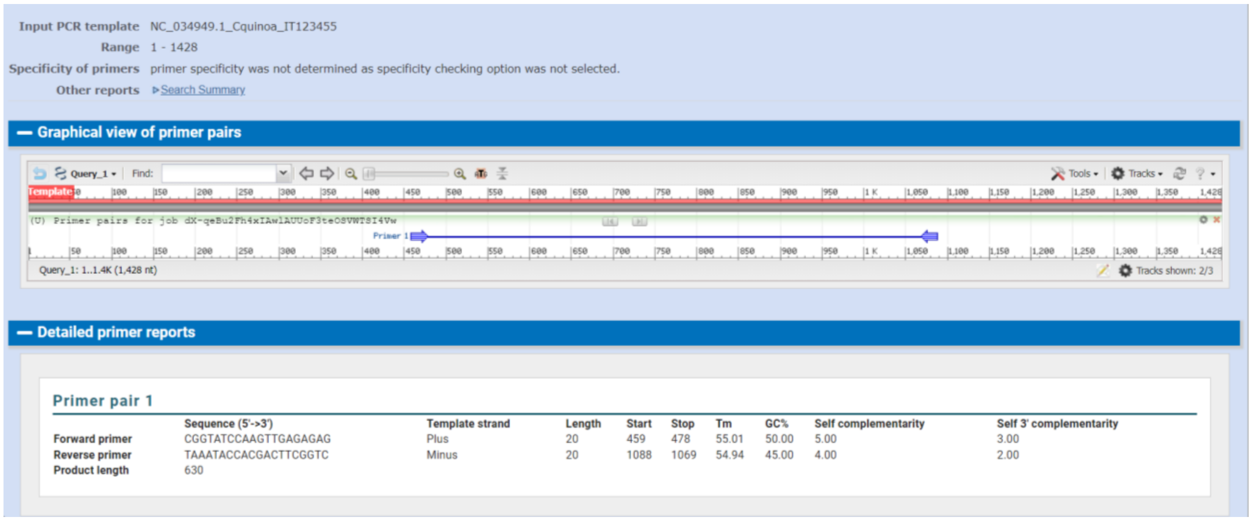

Figure S1. Primer design of rbcL. a, alignment of *rbcL* in *C. quinoa* and *C. album* gene as amplified by primers rbcLM1-F and rbcLM1-R, showing two sites different in the amplicon. b, PCR amplification product of *C. quinoa rbcL* using primers rbcLM1-F and rbcLM1-R, per NCBI Primer-BLAST

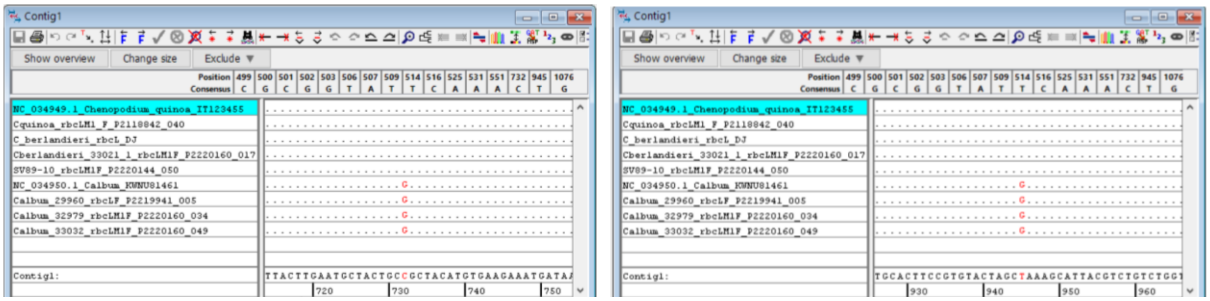

Figure S2. ***rbcL*** **barcoding.** Alignment of references and PCR-amplified DNAs, using primers rbcLM1-F and rbcLM2-R, sequenced in the forward direction using rbcLM1-F (identical sequences on reverse strands). Numbering is from the ATG initiation codon of the *rbcL* gene in the three references. *C. quinoa* and *C. album* reference sequences from NCBI are given with NC_ numbers, *C. berlandieri* reference sequence is from chloroplast genome unpublished, courtesy of David Jarvis. Positive control DNAs of *C. quinoa*, *album* and *berlandieri* were obtained from leaves grown from seeds obtained from David Brenner at the USDA as described in Materials and Methods. Plant SV89-10, collected in Manitoba, Canada, was obtained from Sara Halwas and Anne Worley as described in Materials and Methods. The two sites in *rbcL* different between *C. quinoa* and *C. album* in the amplified region are included.

```
matK_Calbum   361  AAAGAAAAAAAAAGAAATAGTAAAATCTCATAATTTGCGATCTATTCATTCAATATTTCCT
matK_Cquinoa  359  --------AAAAAGAAATAGTAAAATCTCATAATTTGCGATCTATTCATTCAATATTTCCT

matK_Calbum   421  TTTTTAGAGGACAAATTCTTCCATTTCAATTATGTGTTAGATATATTAATAACTTACCCT
matK_Cquinoa  412  TTTTTAGAGGACAAATTCTTCCATTTAAATTATGTGTTAGATATATTAATAACTTACCCT

matK_Calbum   481  GCCCATCTAGAAATCTTGGTTCAAACTCTTCGCTACTGGTTGAAAGATGCCTCTTCTTTG
matK_Cquinoa  472  GCCCATCTAGAAATCTTGGTTCAAACTCTTCGCTACTGGTTGAAAGATGCCTCTTCTTTG

matK_Calbum   541  CATTTAGTAAGATTTTTTCTTTATGAGTCTCGTAATTTAAATAGTCTTATTATTCCAAAG
matK_Cquinoa  532  CATTTAGTAAGATTTTTTCTTTATGAGTCTCGTAATTTAAATAGTCTTATTATTCCAAAG

matK_Calbum   601  GAATTCATTTCCTTTTTGAAAAAAAGGAATCAAAGATTATTCTTGTTCCTATATAATTTC
matK_Cquinoa  592  GAATTCATTTCTTTTTTGAAAAAAAGGAATCAAAGATTATTCTTGTTCCTATATAATTTC

matK_Calbum   661  CATGTATGCGAATACGAATCCTTTTTTGTTTTTCTCCGCAACCAATCCTCTTATTTACGA
matK_Cquinoa  652  CATGTATGCGAATACGAATCTTTTTTTGTTTTTCTCCGCAACCAATCCTCTTATTTACGA

matK_Calbum   721  TCAACCTCTTTTGGAACCCTTATTGAACGAATTTTTTTCTACGGAAAACTAAAATATCTA
matK_Cquinoa  712  TCAACCTCTTTTGGAACCCTTATTGAACGAATTTTTTTCTACGGAAAACTAAAATATTTA

matK_Calbum   781  GTAAAAGTTTTTACTAAGGATTTTGGGGTTATCCTATGGCTTTTCAGAGAACCTTTCCCG
matK_Cquinoa  772  GTAAAAGTTTTTACTAAGGATTTTGGGGTTATCCTATGGCTTTTCAGAGAACCTTTCCCG

matK_Calbum   841  CATTATGTTAGGTATCAAGGAAAATCAATTCTAGCCTCAAAAGGGACATCTCTTTTGATG
matK_Cquinoa  832  CATTATGTTAGGTATCAAGGAAAATCAATTCTAGCCTCAAAAGGGACATCTCTTTTGATG

matK_Calbum   901  CATAAATGGAAAAATTACCTTATCCATTTCTGGCAATGTAATTTTTCTGTGTGGTCTCAA
matK_Cquinoa  892  CATAAATGGAAAAATTACCTTATCCATTTCTGGCAATGTAATTTTTCTGTGTGGTCTCAA

matK_Calbum   961  CCAAAAAGACTCTATATCAATCGATTATCAAACCATTCCTTAGACTTTATGGGTTTTCTT
matK_Cquinoa  952  CCAAAAAGAGTCTATATCAATCGATTATCAAACCATTCCTTAGACTTTATGGGTTTTCTT

matK_Calbum  1021  TCAAATGTGCGACTCAATTCTTCAGTAATACGGAGTCAAATGTTCGAAAATGCATTTCTA
matK_Cquinoa 1012  TCAAATGTGCGACTCAATTCTTCAGTAATACGGAGTCAAATGTTCGAAAATGCATTTCTA

matK_Calbum  1081  ATAGAGAATATTATTAAGAAATTCGATGCCACAGTTCCAATTATTCCTCTGGTTGGATCG
matK_Cquinoa 1072  ATAGAGAATATTATTAAGAAATTCGATGCCACAGTTCCAATTATTCCTCTGGTTGGATCG

matK_Calbum  1141  TTGGCTAAAGCGAAATTTTGTAATGTATTAGGGCATCCCGTTAGTAAGTCGGTCTGGACC
matK_Cquinoa 1132  TTGGCTAAAGCGAAATTTTGTAATGTATTAGGGCATCCCGTTAGTAAGTCGGTCTGGACC

matK_Calbum  1201  GATTTATCCGATTCTGATATTATTGATCGATTTGGACGTATATGTAGAACTATTTTTCAT
matK_Cquinoa 1192  GATTTATCCGATTCTGATATTATTGATCGATTTGGACGTATCTGTAGAACTATTTTTCAT

matK_Calbum  1261  TATTATAGTGGCTCTTCAAGAAAAAAAAAGTTTGTATCGAATAAAGTATATACTTCGATTT
matK_Cquinoa 1252  TATTATAGTGGCTCTTCAAGAAAAAAAAAGTTTGTATCGAATAAAGTATATACTTCGATTT

matK_Calbum  1321  TCTTGTGCTAGAACTTTGTCTCGTAAACACAAAAGTACTGTACGTGCTTTTTTGAAAAGA
matK_Cquinoa 1312  TCTTGTGCTAGAACTTTGTCTCGTAAACACAAAAGTACTGTACGTGCTTTTTTGAAAAGA
```

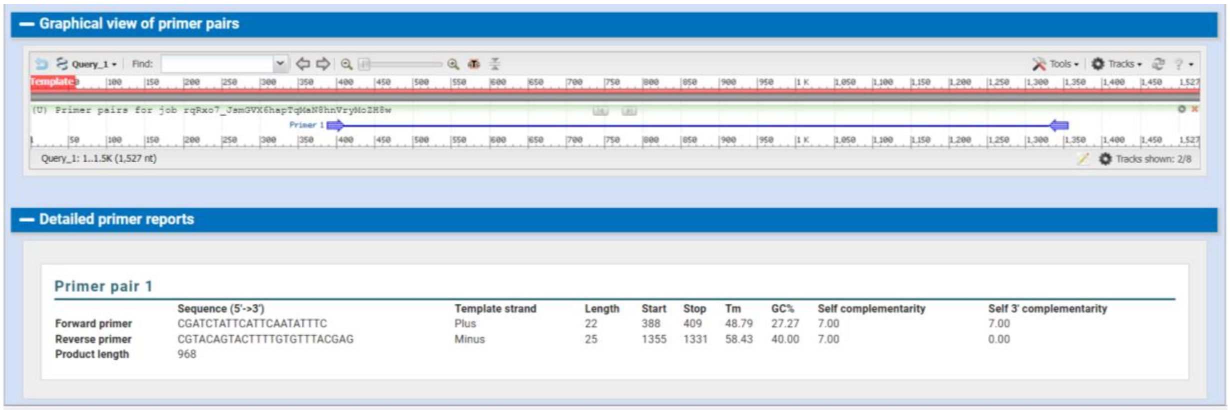

Figure S3. Primer design of matK. a, alignment of *matK* in *C. quinoa* vs *C. album* as amplified by matK390-F and matK3FKIM-R, showing six sites different in the amplicon. b, PCR amplification product of *C. quinoa matK* using primers matK390-F and matK3FK1M-R, per NCBI Primer-BLAST.

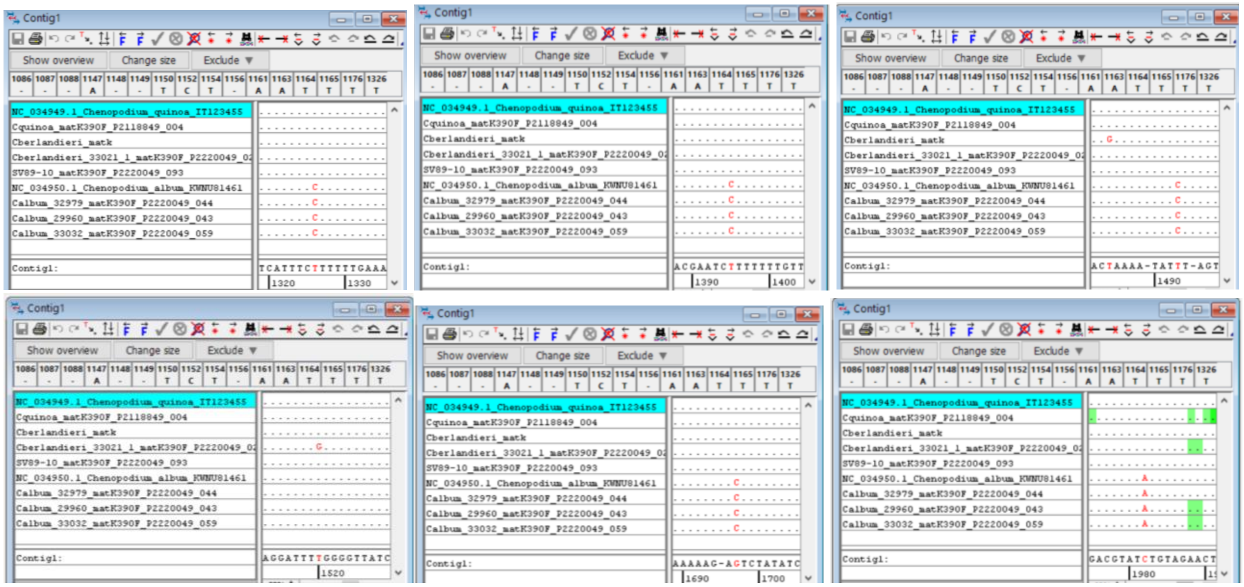

Figure S4. *matK* barcoding. Alignment of references and PCR-amplified DNAs, using primers matK390-F and matK3FKIM-R, sequenced in the forward direction using matK390-F (identical sequences on reverse strands). Numbering is from a position in the *C. berlandieri* chloroplast genome approximately 710 bp upstream of the *matK* ATG initiation codon. *C. quinoa* and *C. album* reference sequences from NCBI are given with NC_ numbers, *C. berlandieri* reference sequence is from chloroplast genome unpublished, courtesy of David Jarvis. Positive control DNAs of *C. quinoa*, *album* and *berlandieri* were obtained from leaves grown from seeds obtained from David Brenner at the USDA as described in Materials and Methods. Plant SV89-10, collected in Manitoba, Canada, was obtained from Sara Halwas and Anne Worley as described in Materials and Methods. Five sites in *matK* different between *C. quinoa* and *C. album* are shown (the sixth different site is too close to one primer for clean sequence), and one site different in the USDA *C. berlandieri* control DNA.

A.

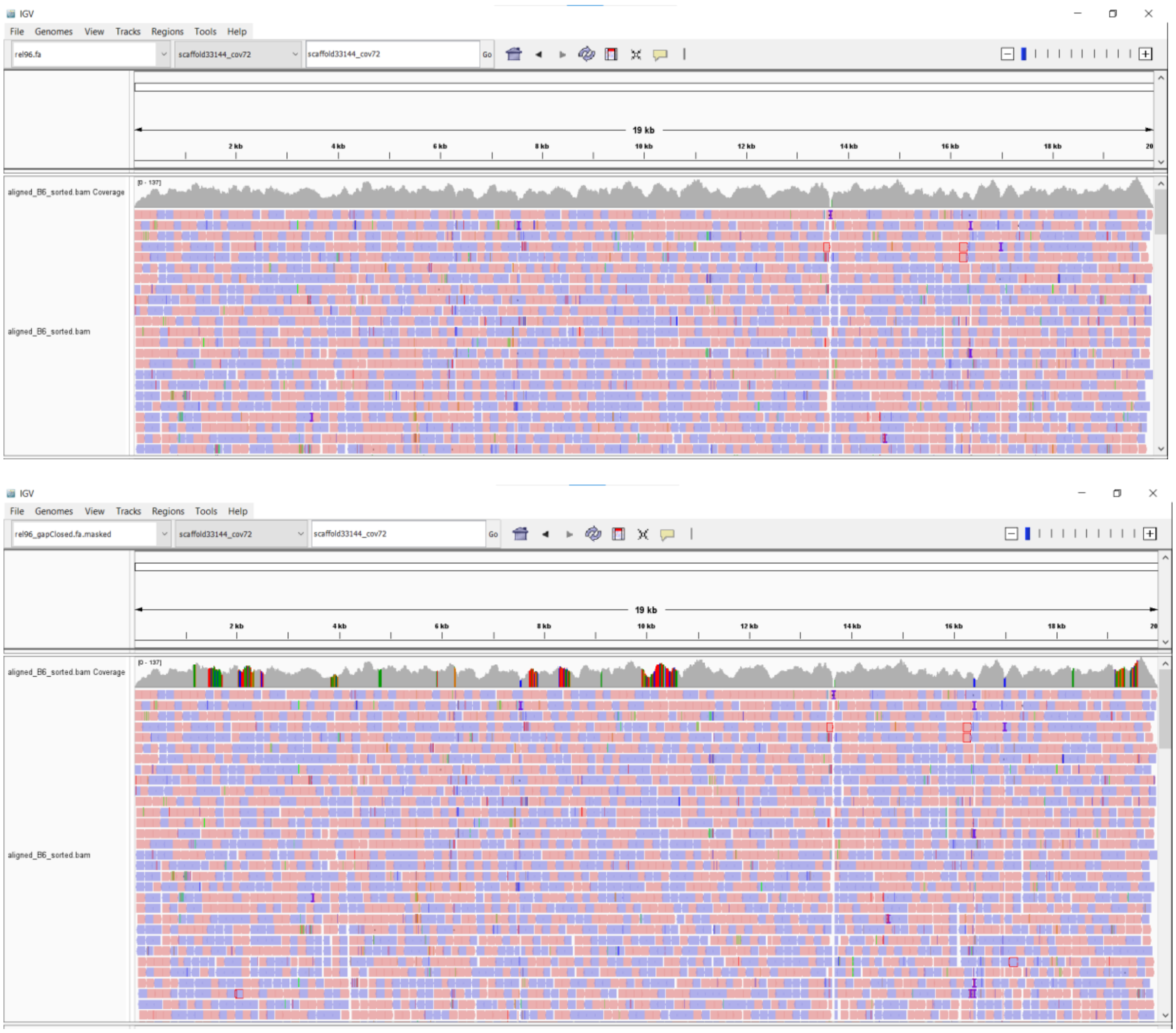
IGV
File Genomes View Tracks Regions Tools Help
rel96.fa
scaffold33144_cov72
scaffold33144_cov72
Go
19 kb
2 kb
4 kb
6 kb
8 kb
10 kb
12 kb
14 kb
16 kb
18 kb
aligned_B6_sorted.bam Coverage
aligned_B6_sorted.bam
IGV
File Genomes View Tracks Regions Tools Help
rel96_gapClosed.fa.masked
scaffold33144_cov72
scaffold33144_cov72
Go
19 kb
aligned_B6_sorted.bam Coverage
aligned_B6_sorted.bam

B.

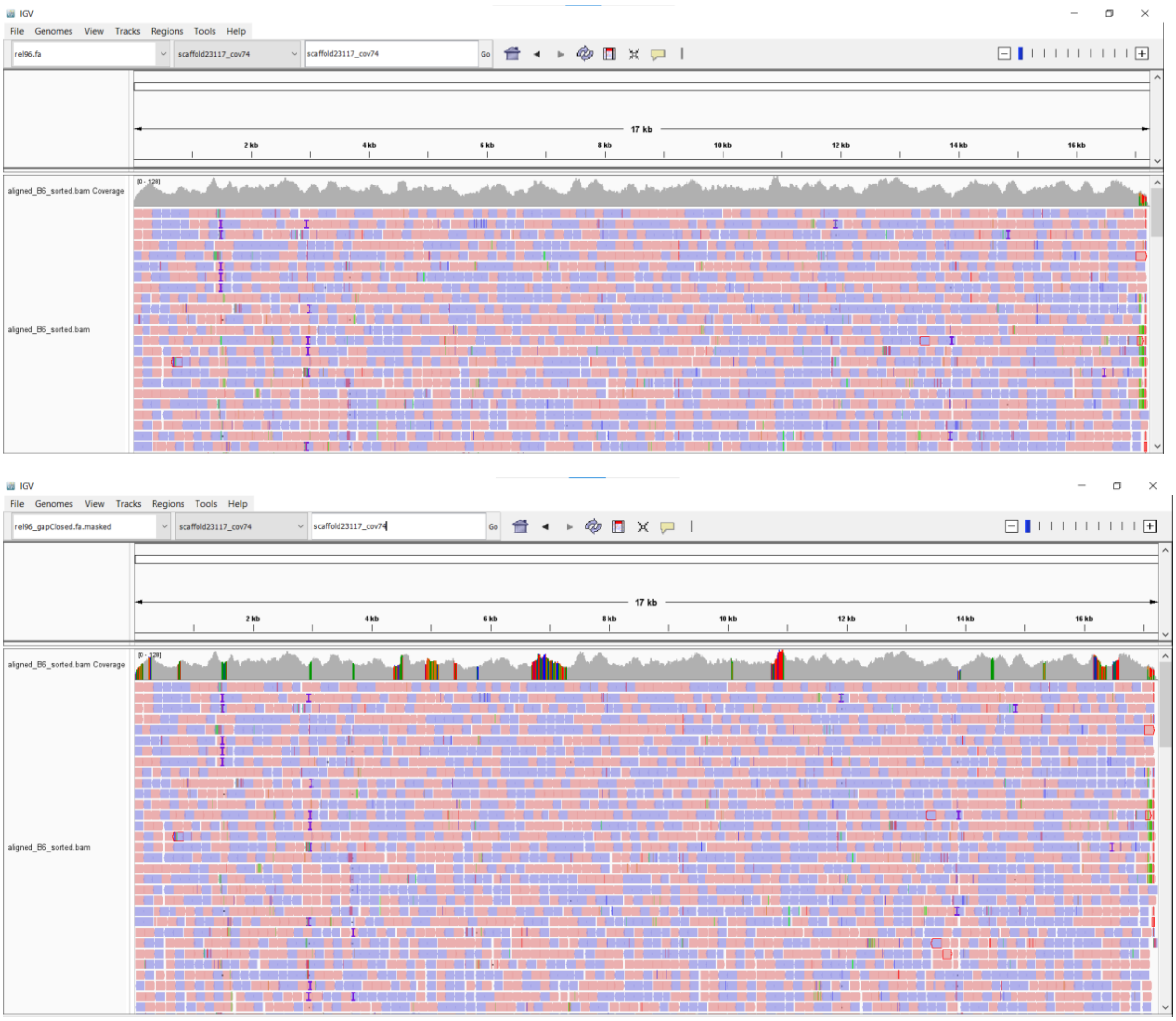
IGV
File Genomes View Tracks Regions Tools Help
rel96.fa
scaffold23117_cov74
scaffold23117_cov74
17 kb
2 kb
4 kb
6 kb
8 kb
10 kb
12 kb
14 kb
16 kb
aligned_B6_sorted.bam Coverage
aligned_B6_sorted.bam
IGV
File Genomes View Tracks Regions Tools Help
rel96_gapClosed.fa.masked
scaffold23117_cov74
scaffold23117_cov74
17 kb
2 kb
4 kb
6 kb
8 kb
10 kb
12 kb
14 kb
16 kb
aligned_B6_sorted.bam Coverage
aligned_B6_sorted.bam

C.

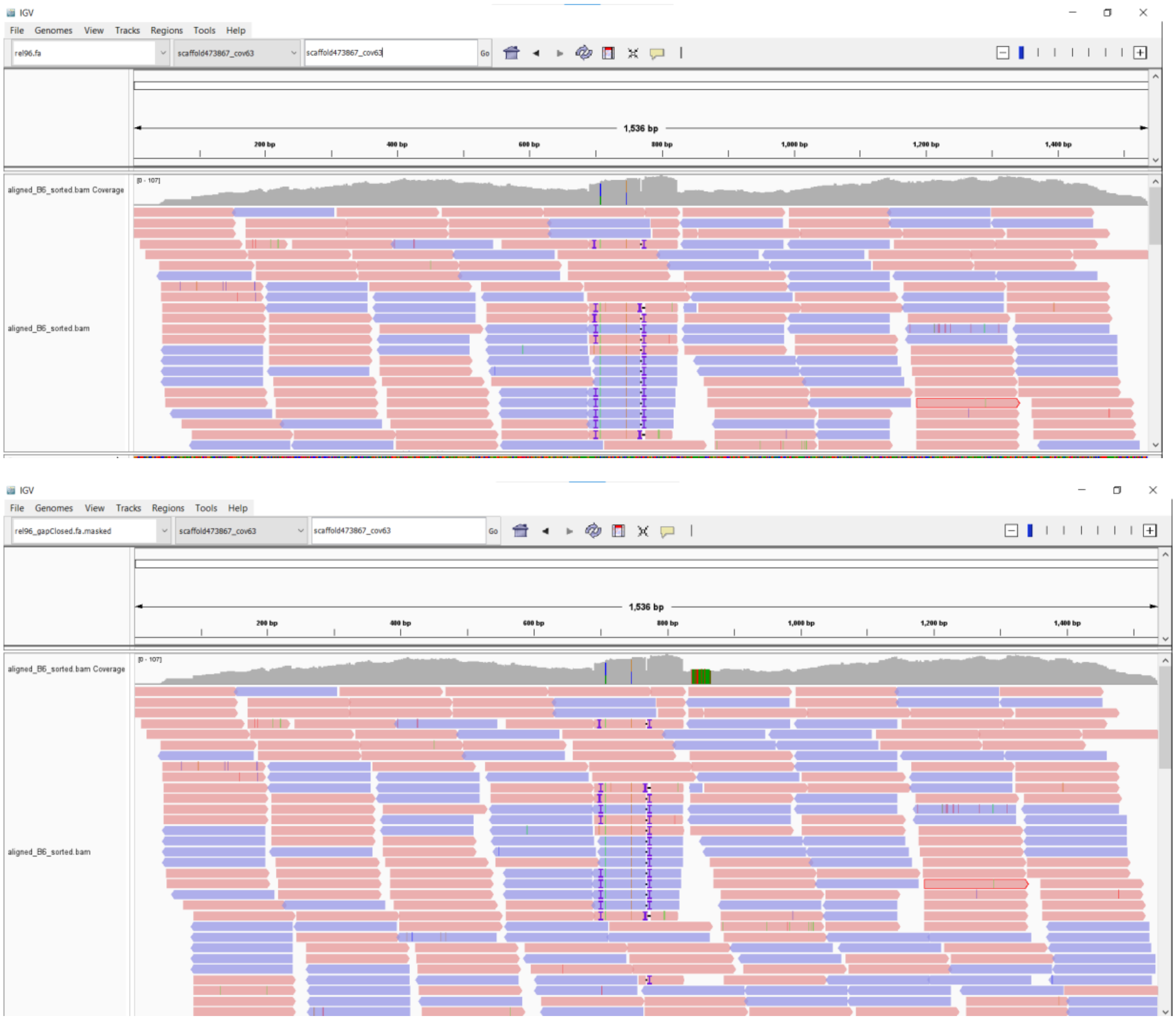
IGV
File Genomes View Tracks Regions Tools Help
rel96.fa
scaffold473867_cov63
Go
1,536 bp
200 bp
400 bp
600 bp
800 bp
1,000 bp
1,200 bp
1,400 bp
aligned_B6_sorted.bam Coverage
[0 - 107]
aligned_B6_sorted.bam
IGV
File Genomes View Tracks Regions Tools Help
rel96_gapClosed.fa.masked
scaffold473867_cov63
Go
1,536 bp
200 bp
400 bp
600 bp
800 bp
1,000 bp
1,200 bp
1,400 bp
aligned_B6_sorted.bam Coverage
[0 - 107]
aligned_B6_sorted.bam

D.

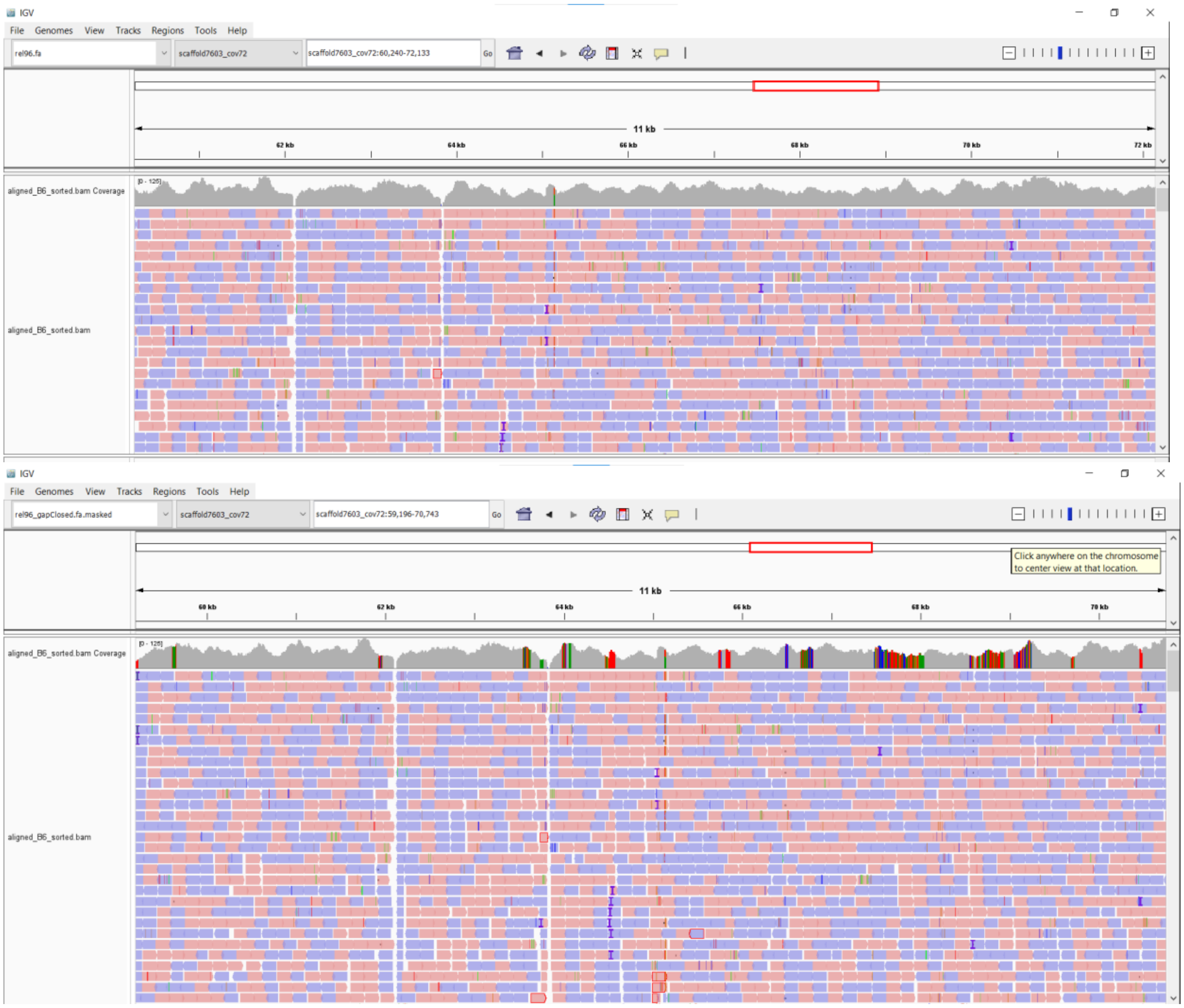
IGV
File Genomes View Tracks Regions Tools Help
rel96.fa
scaffold7603_cov72
scaffold7603_cov72:60,240-72,133
11 kb
aligned_B6_sorted.bam Coverage
aligned_B6_sorted.bam
IGV
File Genomes View Tracks Regions Tools Help
rel96_gapClosed.fa.masked
scaffold7603_cov72
scaffold7603_cov72:59,196-70,743
Click anywhere on the chromosome to center view at that location.
11 kb
aligned_B6_sorted.bam Coverage
aligned_B6_sorted.bam

E.

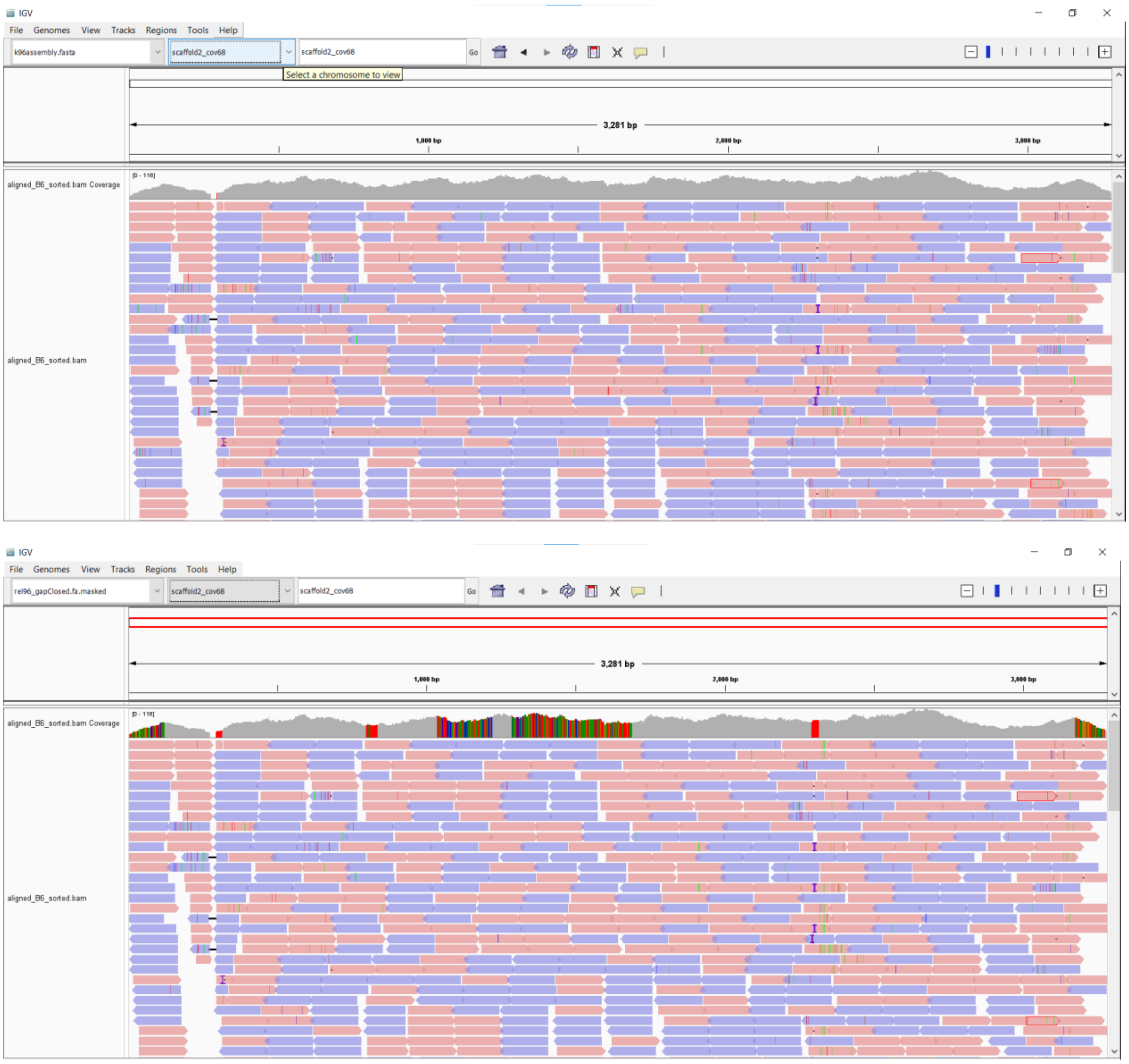
IGV
File Genomes View Tracks Regions Tools Help
k96assembly.fasta
scaffold2_cov68
scaffold2_cov68
Go
Select a chromosome to view
3,281 bp
1,000 bp
2,000 bp
3,000 bp
aligned_B6_sorted.bam Coverage
[0 - 116]
aligned_B6_sorted.bam
IGV
File Genomes View Tracks Regions Tools Help
rel96_gapClosed.fa.masked
scaffold2_cov68
scaffold2_cov68
Go
3,281 bp
1,000 bp
2,000 bp
3,000 bp
aligned_B6_sorted.bam Coverage
[0 - 116]
aligned_B6_sorted.bam

F.

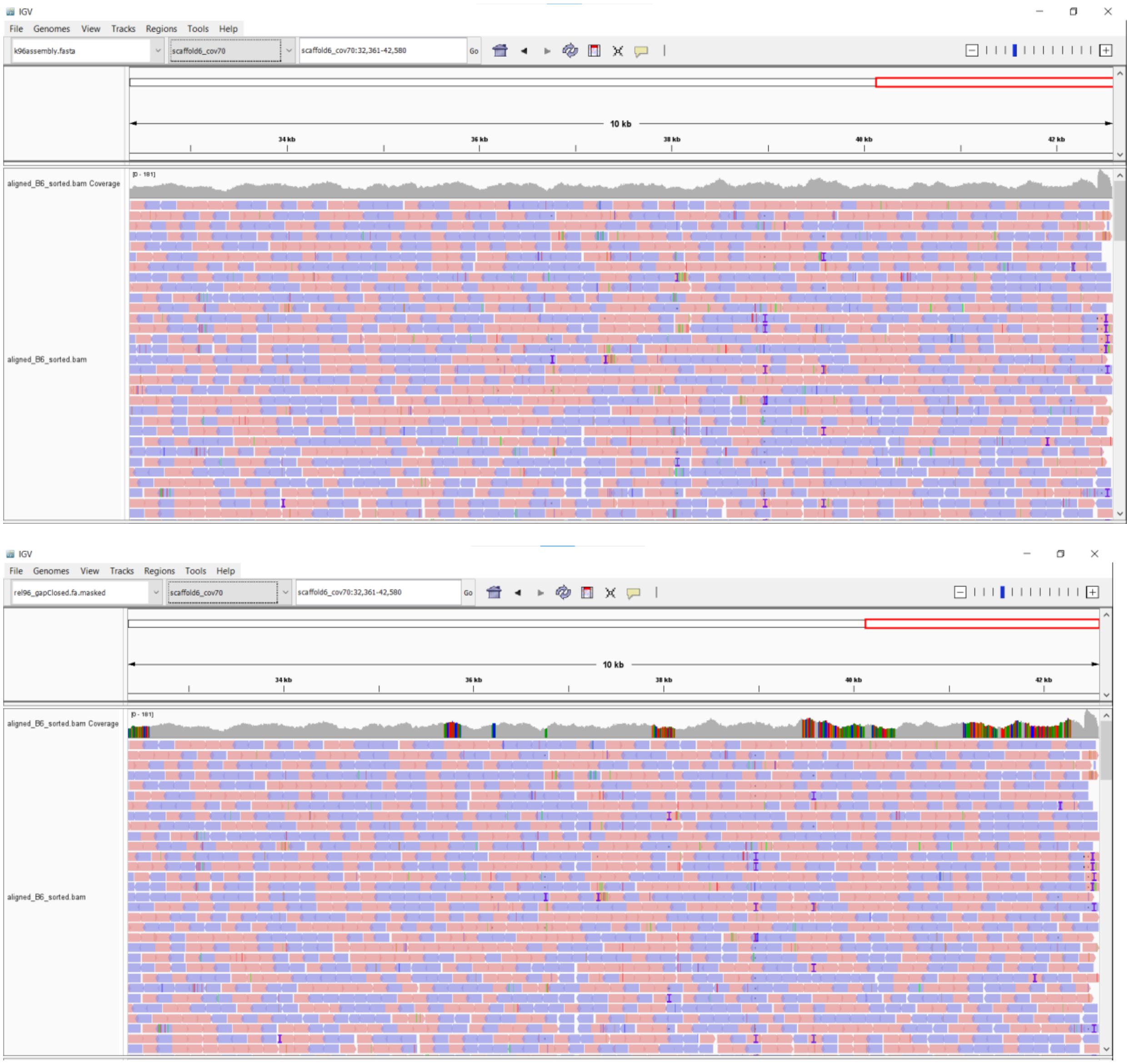

Figure S5. Selected scaffolds showing examples of heterozygous SNPs. Each scaffold is shown twice, first aligned to the whole genome assembly, then below aligned to the genome assembly with repeats masked. Split vertical colored lines present in both alignments of the scaffold represent heterozygous sites. Solid vertical colored lines present in only the lower alignment represent repetitive regions (where the bases called in the scaffold are different from the N's in the masked genome assembly, hence are shown as solid colored lines). A-C, scaffolds of *SOS1* homologs: A, scaffold33144; B, scaffold23117; C, scaffold473876. D-F, miscellaneous scaffolds: D, scaffold7603; E, scaffold2; F, scaffold6.

A.

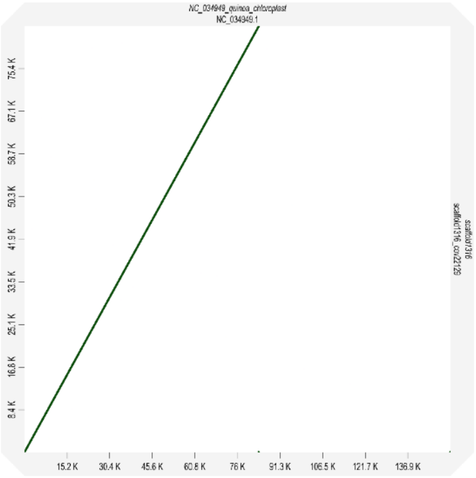

B.

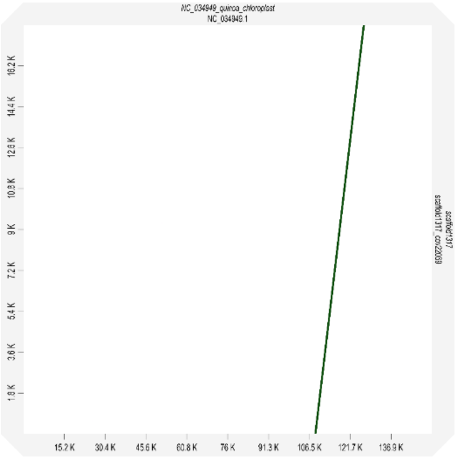

C.

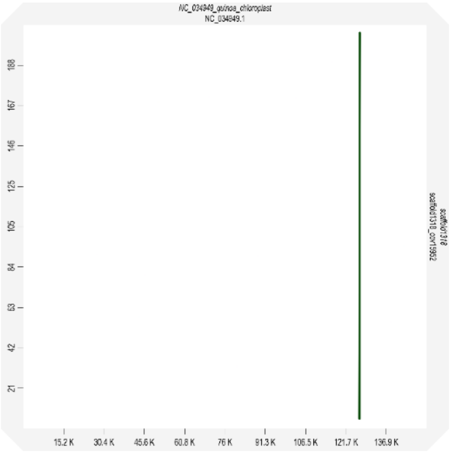

Figure S6. Chloroplast genome assembly. A, B, C, alignments to *C. quinoa* unique chloroplast genome regions.

A.

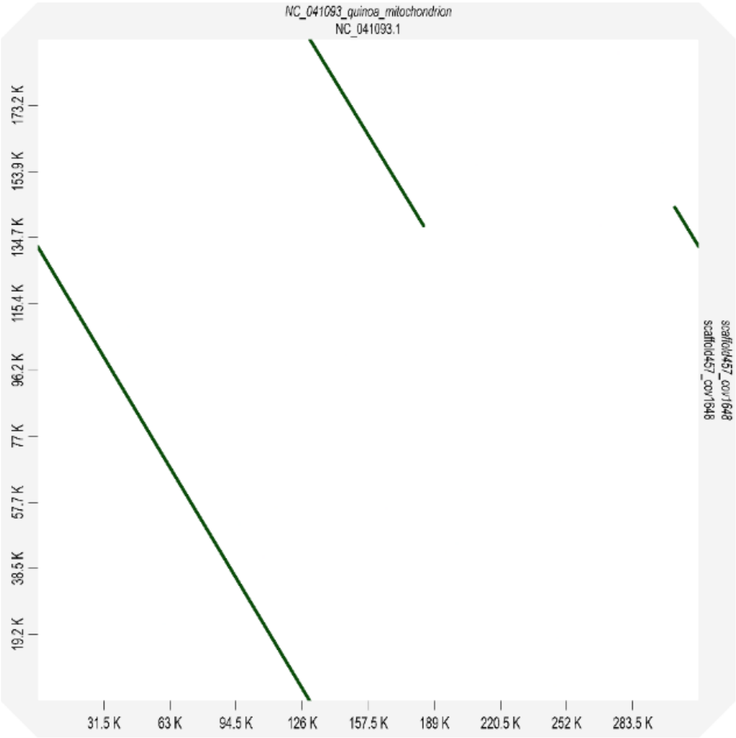

B.

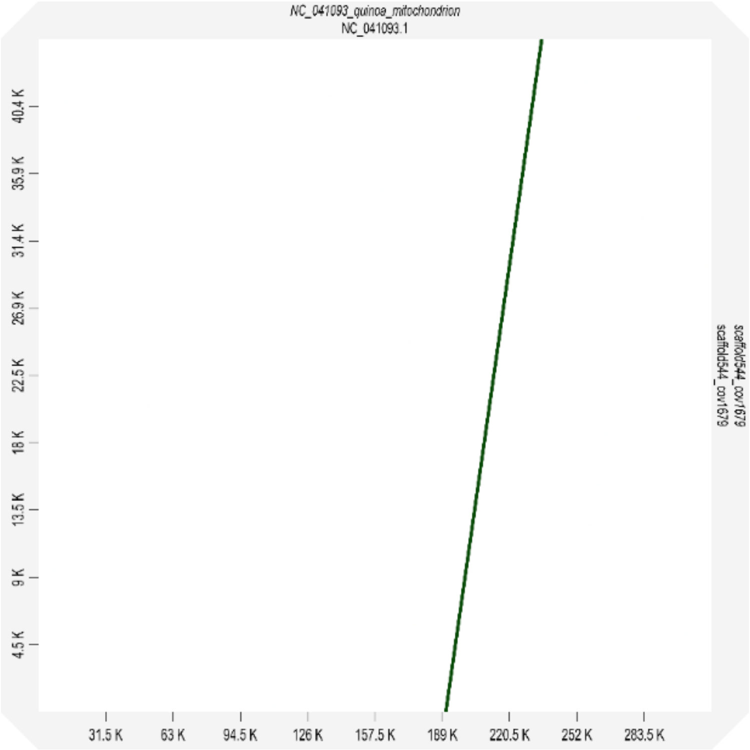

Figure S7. Mitochondrial genome assembly. A, B, alignments to *C. quinoa* mitochondrial genome.

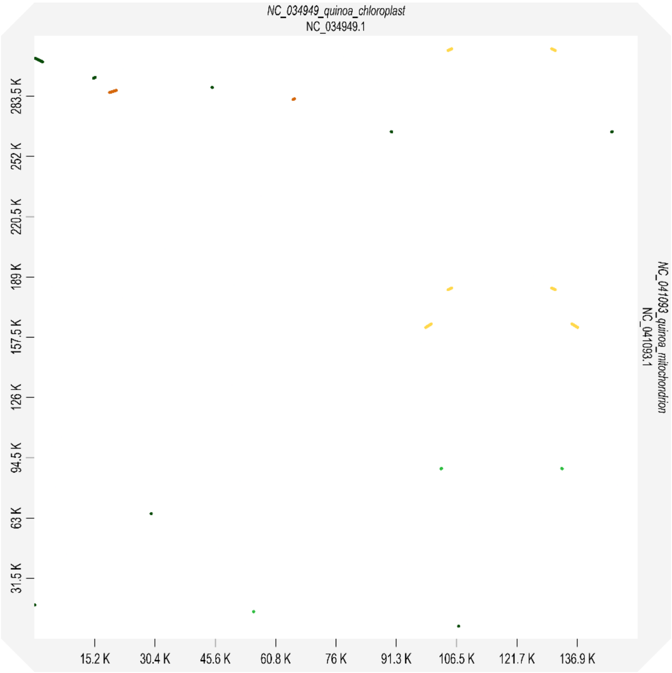

Figure S8. Alignment of *C. quinoa* chloroplast and mitochondrial genomes to each other.

A.

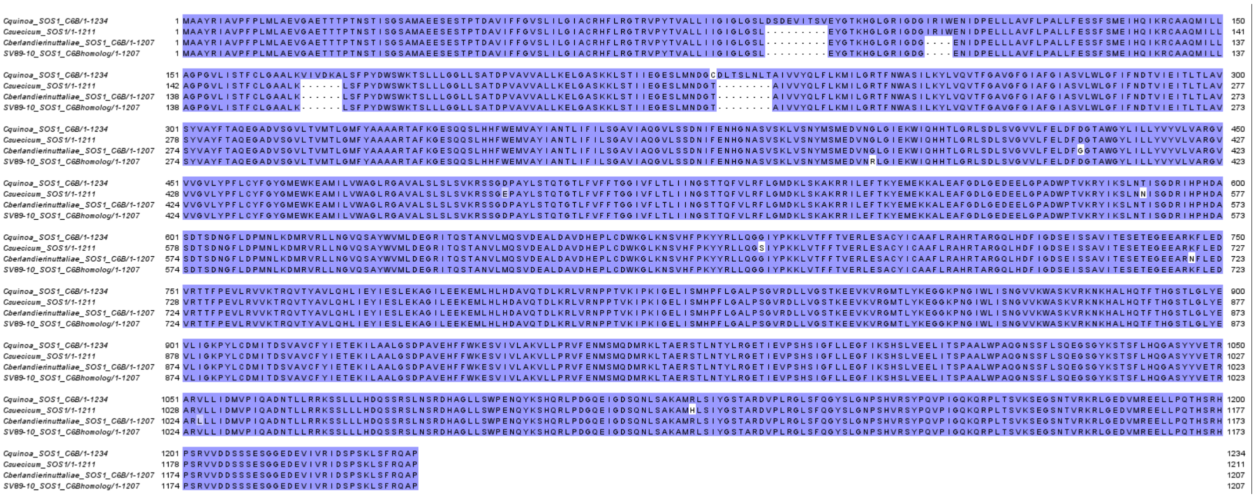

B.

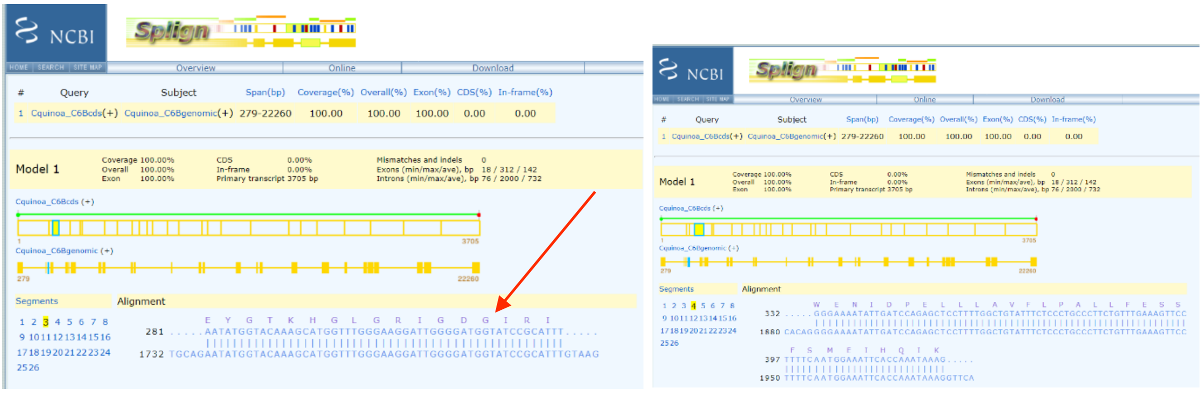
NCBI
Splign
Overview
Online
Download
Model 1
Segments
Alignment

C.

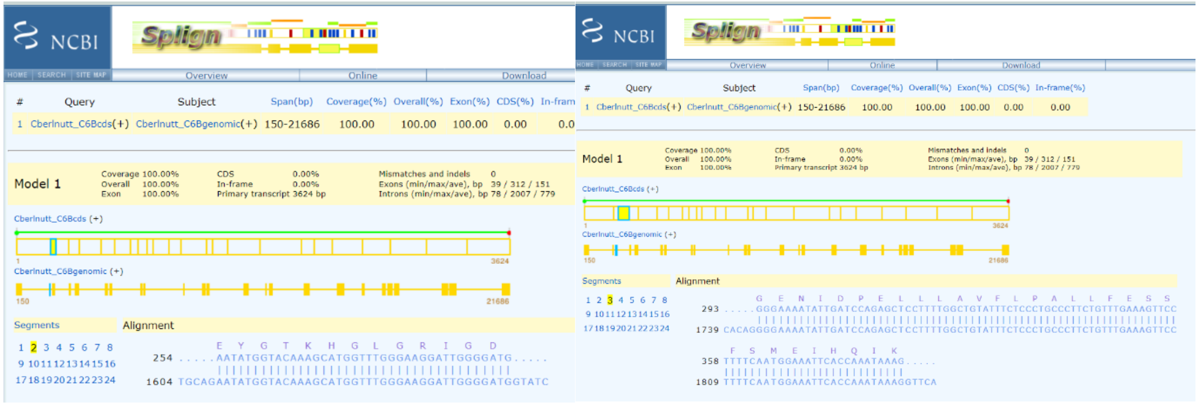
NCBI
Splign
Overview
Online
Download
Model 1
Segments
Alignment

D.

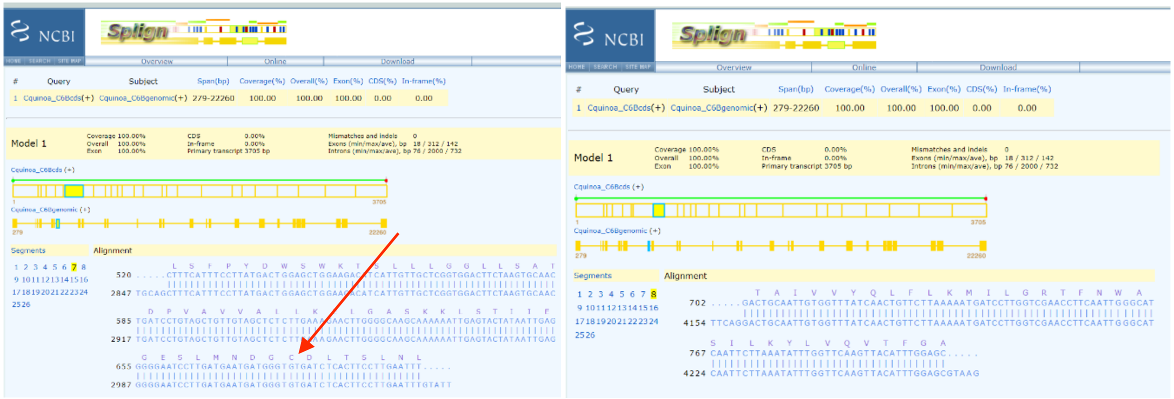

E.

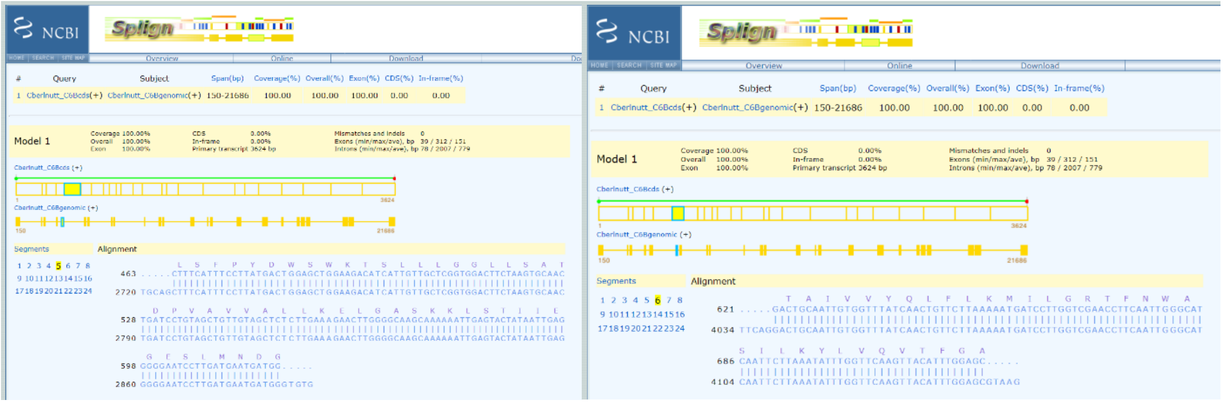

F.

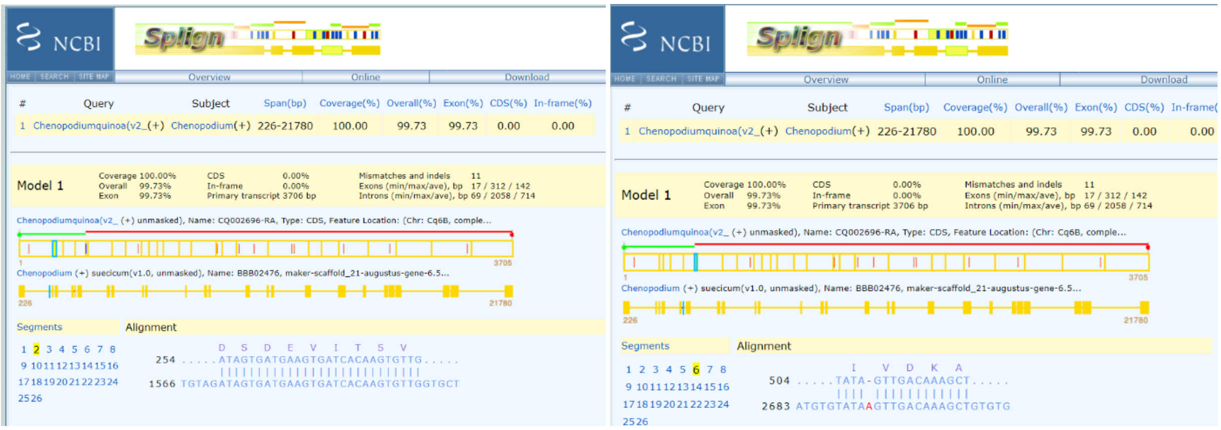

Figure S9. Gene models of *SOS1* sub-genome B. In all panels, the sequence whose name begins SV89-10 is from the *de novo* assembly reported here. A. Multiple sequence alignment of gene models from CoGe database for *C. quinoa* sub-genome B, *C. berlandieri nuttaliae,* and *C. suecicum* (the sub-genome B

related diploid), with the gene model assembled from our assembly scaffolds with greatest similarity to the quinoa sub-genome B homolog. B. *C. quinoa SOS1* C6B homolog, exon 3/4 junction, red arrow indicates GT splice donor site used in other species gene annotations. C. *C. berlandieri nuttaliae SOS1* C6B homolog exon 2/3 junction. D. *C. quinoa SOS1* C6B homolog, exon 7/8 junction, red arrow indicates GT splice donor site used in other species gene annotations. E. *C. berlandieri nuttaliae SOS1* C6B homolog, exon 6/7 junction. F. Possible alternative exons in *C. suecicum* genomic sequence, using the *C. quinoa SOS1* C6B homolog as reference.

A.

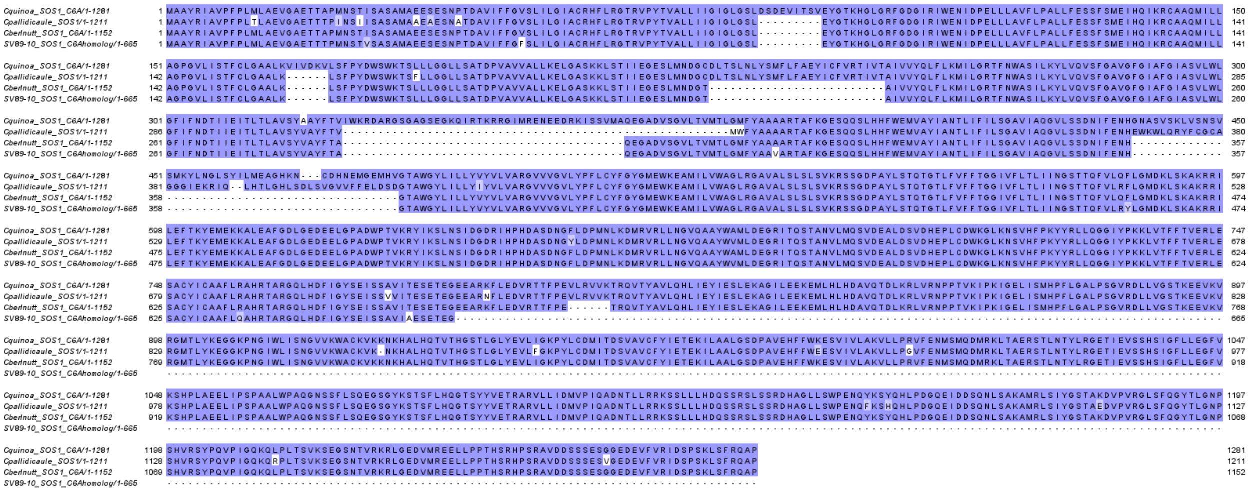

B.

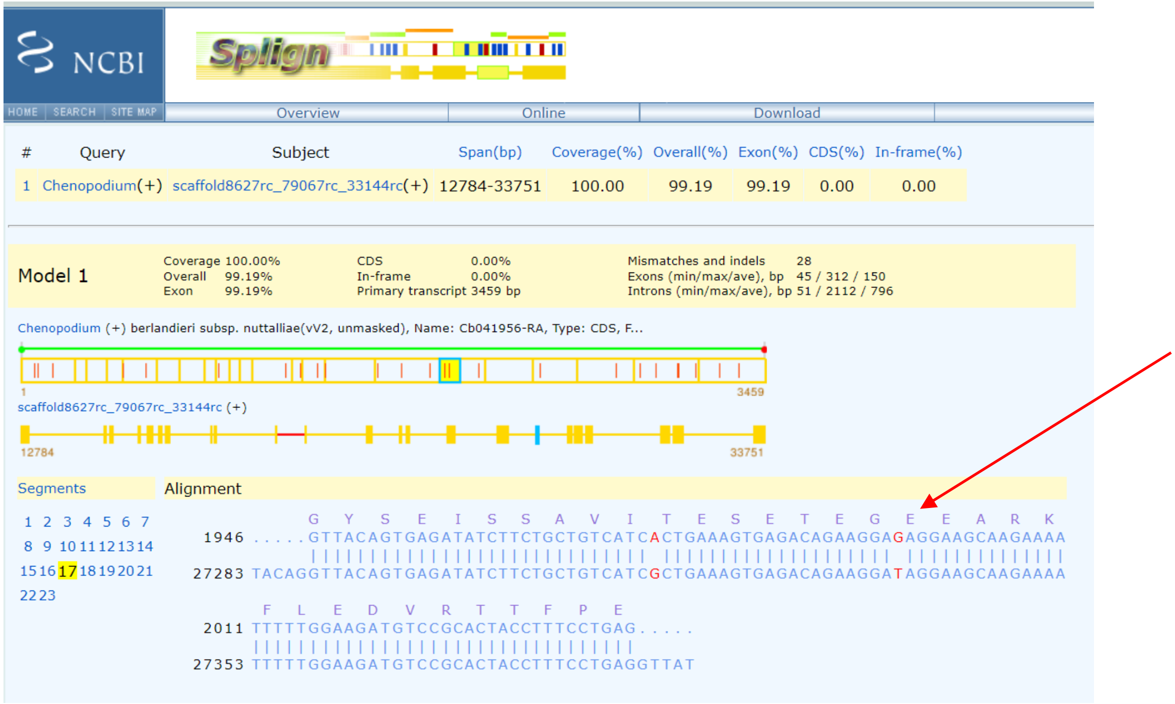
NCBI
Splign
HOME SEARCH SITE MAP
Overview
Online
Download
Query Subject Span(bp) Coverage(%) Overall(%) Exon(%) CDS(%) In-frame(%)
1 Chenopodium(+) scaffold8627rc_79067rc_33144rc(+) 12784-33751 100.00 99.19 99.19 0.00 0.00
Model 1
Coverage 100.00%
Overall 99.19%
Exon 99.19%
CDS 0.00%
In-frame 0.00%
Primary transcript 3459 bp
Mismatches and indels 28
Exons (min/max/ave), bp 45 / 312 / 150
Introns (min/max/ave), bp 51 / 2112 / 796
Chenopodium (+) berlandieri subsp. nuttalliae(vV2, unmasked), Name: Cb041956-RA, Type: CDS, F...
1
3459
scaffold8627rc_79067rc_33144rc (+)
12784
33751
Segments
Alignment
1 2 3 4 5 6 7
8 9 10 11 12 13 14
15 16 17 18 19 20 21
22 23
G Y S E I S S A V I T E S E T E G E E A R K
1946 .....GTTACAGTGAGATATCTTCTGCTGTCATCACTGAAAGTGAGACAGAAGGAGAGGAAGCAAGAAAA
27283 TACAGGTTACAGTGAGATATCTTCTGCTGTCATCGCTGAAAGTGAGACAGAAGGATAGGAAGCAAGAAAA
F L E D V R T T F P E
2011 TTTTTGGAAGATGTCCGCACTACCTTTCCTGAG.....
27353 TTTTTGGAAGATGTCCGCACTACCTTTCCTGAGGTTAT

C.

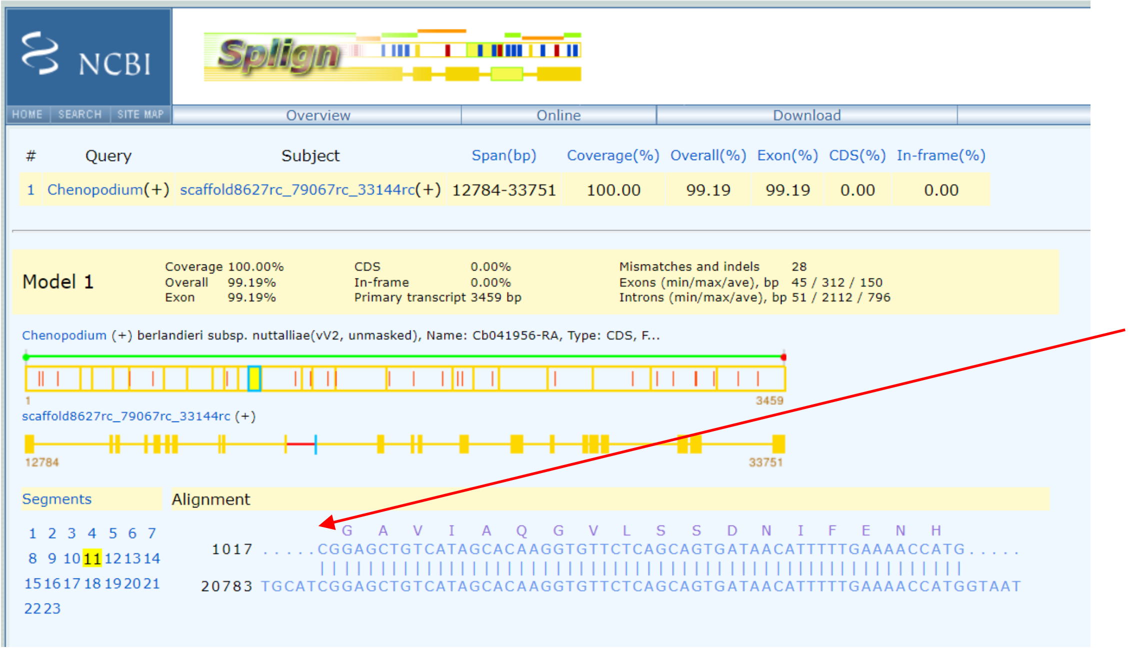

Figure S10. Gene models of *SOS1* sub-genome A. In all panels, the sequence whose name begins SV89-10 is from the *de novo* assembly reported here. A. Multiple sequence alignment of gene models from CoGe database for *C. quinoa* sub-genome A, *C. berlandieri nuttaliae,* and *C. pallidicaule* (the sub-genome A related diploid), with the gene model created from our assembly scaffolds with greatest similarity to the quinoa sub-genome A homolog. B. *C. berlandieri nuttaliae SOS1* homolog C6A coding sequence aligned to *C. berlandieri de novo* assembly, exon 17. In frame stop codon in *de novo* assembly indicated with red arrow. C. *C. berlandieri nuttaliae SOS1* homolog C6A coding sequence aligned to *C. berlandieri de novo* assembly, exon 11. Mutation in splice acceptor site in de novo assembly indicated with red arrow.

A.

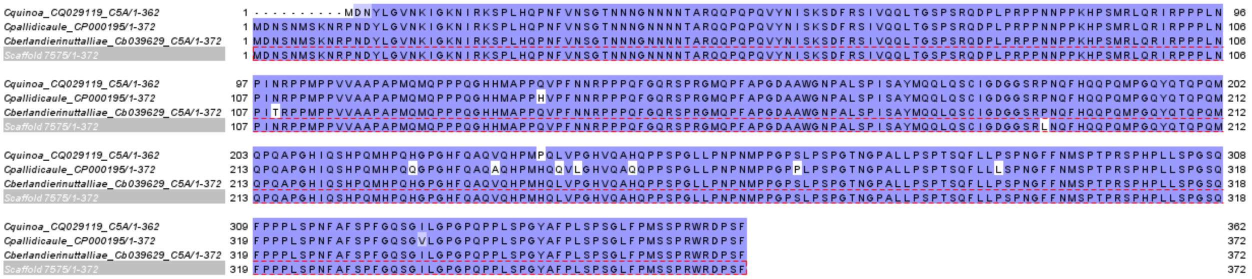

B.

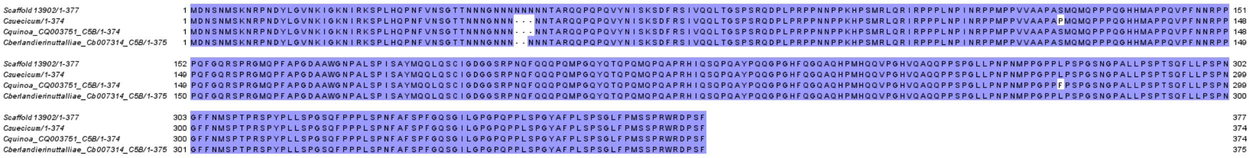

Figure S11. *IKU1* multiple sequence alignment. A. Homologs of quinoa *IKU1* sub-genome A. B. Homologs of quinoa *IKU1* sub-genome B. In both panels, the sequences whose names begin with 'Scaffold' are those of the *de novo* assembly reported here.

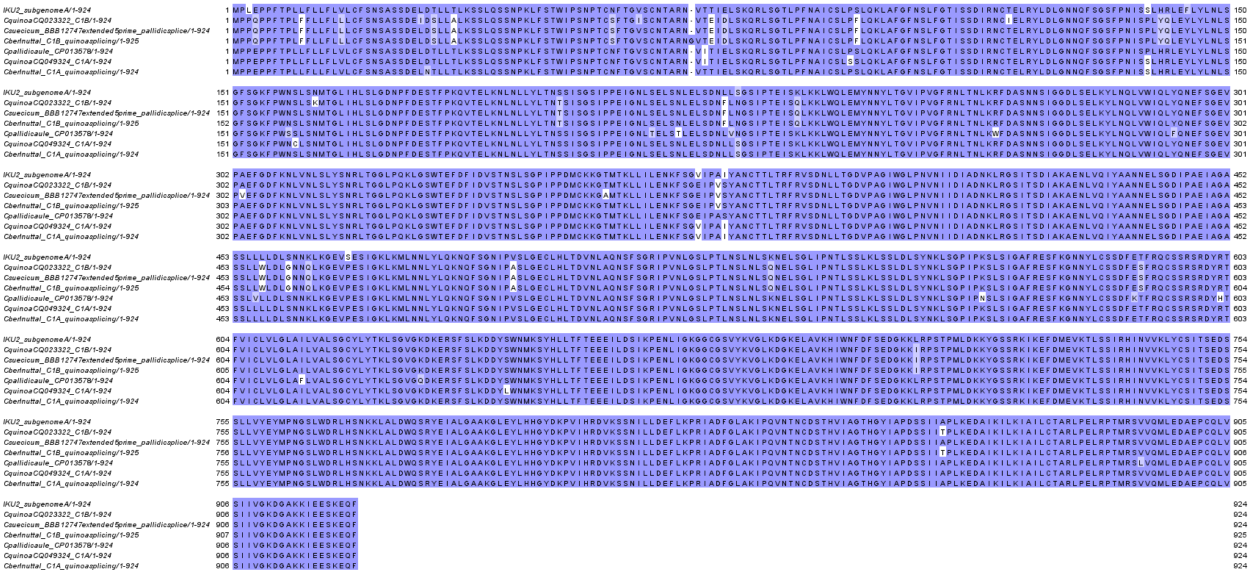

Figure S12. *IKU2* gene models. As described in the text, splice sites and initiating methionines were used for maximum consistency in all the models, although in several cases the genes are differently annotated in the CoGe database. The sequence whose name begins IKU2_sub-genomeA is that of the assembly reported here.

A.

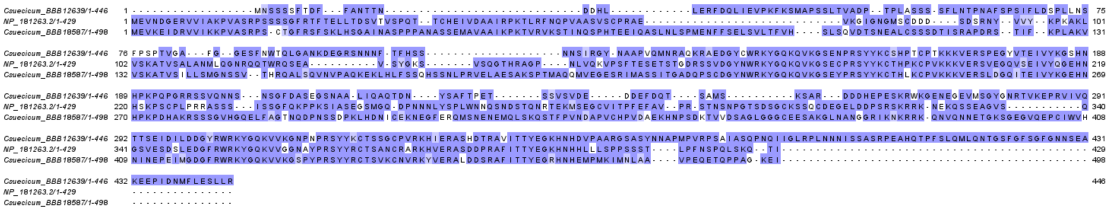

B.

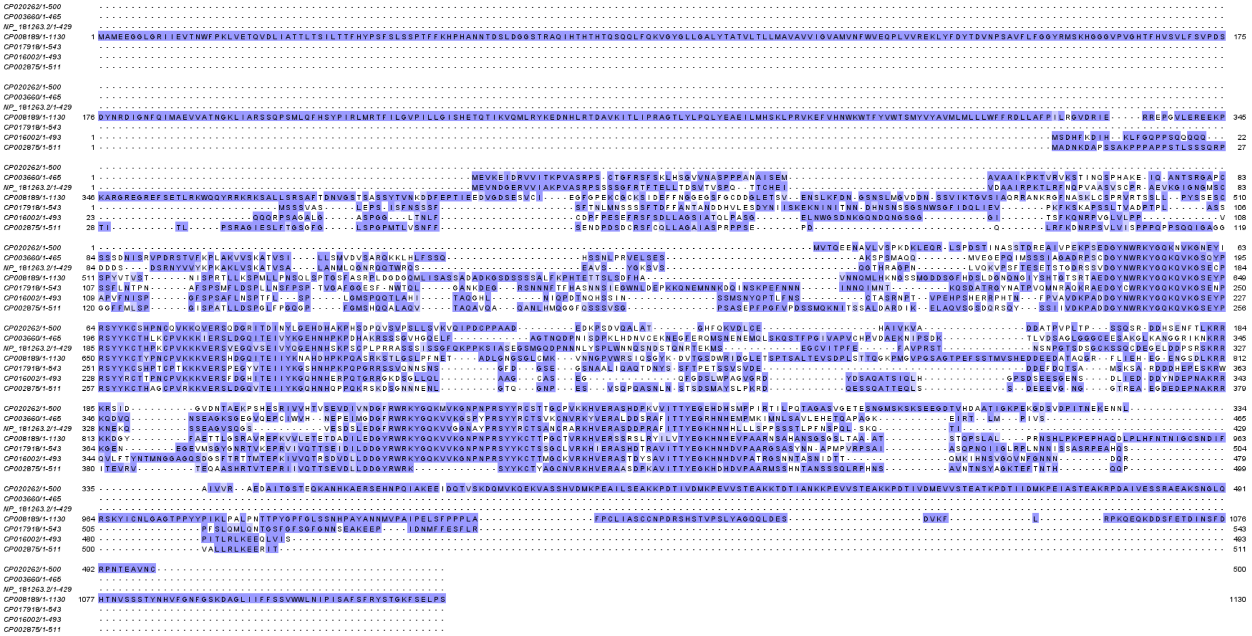

C.

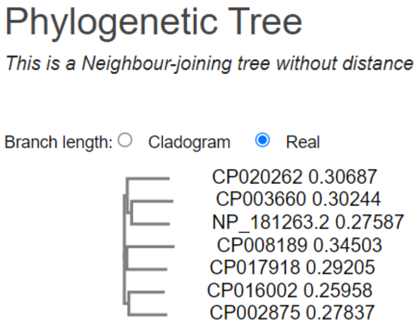
Phylogenetic Tree
This is a Neighbour-joining tree without distance corrections.
Branch length: Cladogram Real
CP020262 0.30687
CP003660 0.30244
NP_181263.2 0.27587
CP008189 0.34503
CP017918 0.29205
CP016002 0.25958
CP002875 0.27837

D.

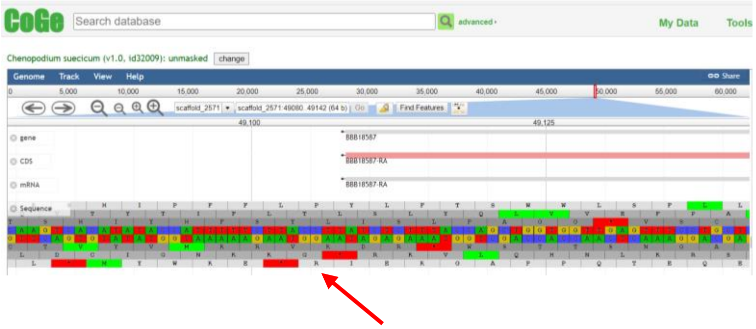
CoGe
Search database
My Data
Tools

E.

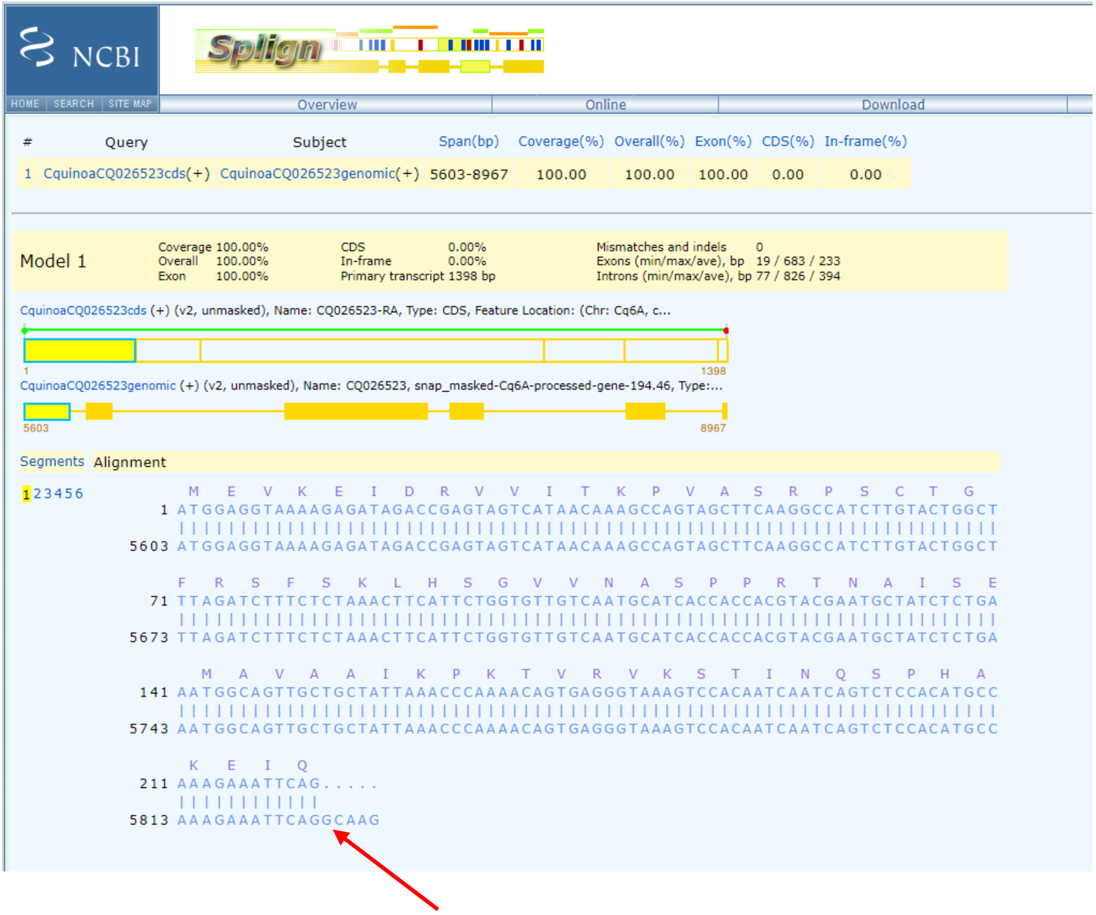
NCBI
Splign
HOME SEARCH SITE MAP
Overview
Online
Download
Query Subject Span(bp) Coverage(%) Overall(%) Exon(%) CDS(%) In-frame(%)
1 CquinoaCQ026523cds(+) CquinoaCQ026523genomic(+) 5603-8967 100.00 100.00 100.00 0.00 0.00
Model 1
Coverage 100.00%
Overall 100.00%
Exon 100.00%
CDS 0.00%
In-frame 0.00%
Primary transcript 1398 bp
Mismatches and indels 0
Exons (min/max/ave), bp 19 / 683 / 233
Introns (min/max/ave), bp 77 / 826 / 394
CquinoaCQ026523cds (+) (v2, unmasked), Name: CQ026523-RA, Type: CDS, Feature Location: (Chr: Cq6A, c...
1
1398
CquinoaCQ026523genomic (+) (v2, unmasked), Name: CQ026523, snap_masked-Cq6A-processed-gene-194.46, Type:...
5603
8967
Segments Alignment
123456
M E V K E I D R V V I T K P V A S R P S C T G
1 ATGGAGGTAAAAGAGATAGACCGAGTAGTCATAACAAAGCCAGTAGCTTCAAGGCCATCTTGTACTGGCT
5603 ATGGAGGTAAAAGAGATAGACCGAGTAGTCATAACAAAGCCAGTAGCTTCAAGGCCATCTTGTACTGGCT
F R S F S K L H S G V V N A S P P R T N A I S E
71 TTAGATCTTTCTCTAAACTTCATTCTGGTGTTGTCAATGCATCACCACCACGTACGAATGCTATCTCTGA
5673 TTAGATCTTTCTCTAAACTTCATTCTGGTGTTGTCAATGCATCACCACCACGTACGAATGCTATCTCTGA
M A V A A I K P K T V R V K S T I N Q S P H A
141 AATGGCAGTTGCTGCTATTAAACCCAAAACAGTGAGGGTAAAGTCCACAATCAATCAGTCTCCACATGCC
5743 AATGGCAGTTGCTGCTATTAAACCCAAAACAGTGAGGGTAAAGTCCACAATCAATCAGTCTCCACATGCC
K E I Q
211 AAAGAAATTCAG.....
5813 AAAGAAATTCAGGCAAG

F.

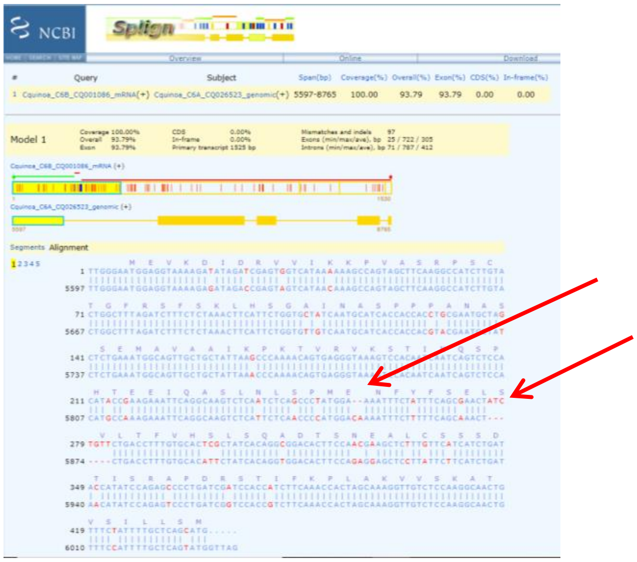
NCBI
Splign
Model 1

G.

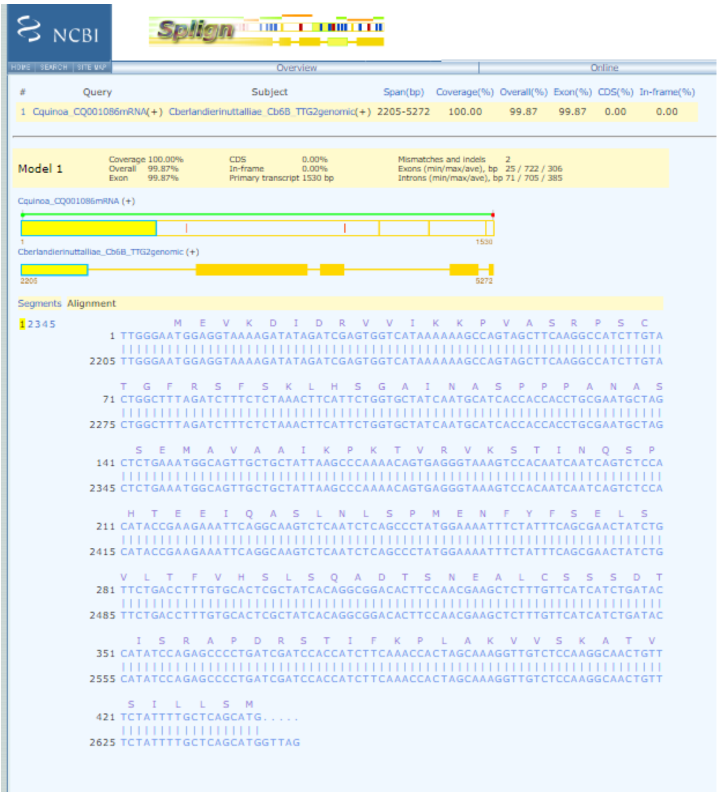
NCBI
Splign
Overview
Online
Query Subject Span(bp) Coverage(%) Overall(%) Exon(%) CDS(%) In-frame(%)
1 Cquinoa_CQ001086mRNA(+) Cberlandierinuttalliae_Cb6B_TTG2genomic(+) 2205-5272 100.00 99.87 99.87 0.00 0.00
Model 1
Cquinoa_CQ001086mRNA (+)
Cberlandierinuttalliae_Cb6B_TTG2genomic (+)
Segments Alignment

H.

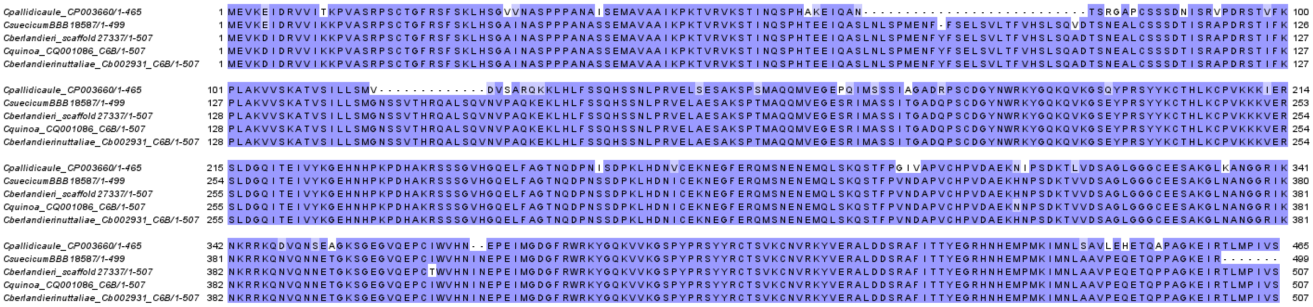
Cpallidicaule_CP003660/1-465
CsuecicumBBB18587/1-499
Cberlandieri_scaffold27337/1-507
Cquinoa_CQ001086_C6B/1-507
Cberlandierinuttaliae_Cb002931_C6B/1-507

I.

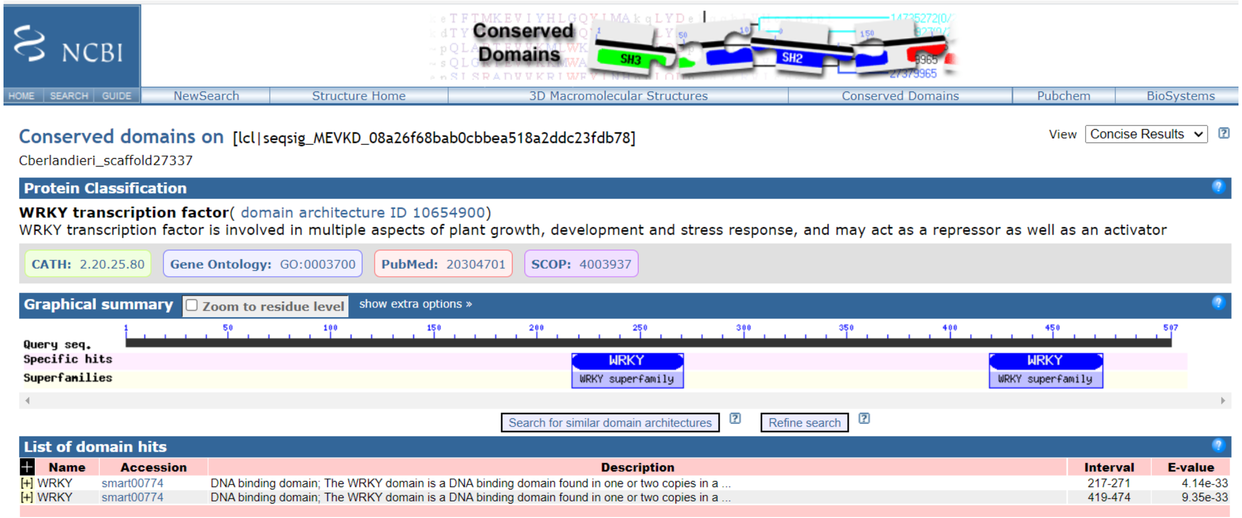

Figure S13. *TTG2* gene models. A. Potential homologs of *A. thaliana TTG2* (NP_181263) in *C. suecicum.* B. Potential homologs of *A. thaliana TTG2* (NP_181263) in *C.* pallidicaule. C. Clustal Phylogeny derived tree for potential homologs of *TTG2* in *C. pallidicaule*. D. C-terminus of *C. suecicum* homolog, showing complete open reading frame extending one amino acid further (red arrow) than the annotated protein in the database itself (n.b. reading frame goes from right to left in this window as gene is on bottom strand of the genomic scaffold as annotated). The extra C-terminal R is included in the alignment in panel E. E. Splign alignment of annotated *C. quinoa* CDS (upper aligned sequence) to genomic (lower aligned sequence) for chromosome 6A gene CQ026523, showing GC splice donor site of first intron. F. Splign alignment of C. quinoa chromosome 6B gene CQ001086 mRNA (upper aligned sequence) to chromosome 6A CQ026523 genomic (lower aligned sequence). Red arrows indicate positions of a 2bp insertion and a 7 bp deletion in the genomic sequence, whose net effect is to create a frameshift, severely truncating the correct open reading frame. G. Splign alignment of *C. quinoa* chromosome 6B gene CQ001086 mRNA (upper aligned sequence) to chromosome 6B sequence of *C. berlandieri nuttaliae* extended downstream of annotated 3’ end of gene Cb002931; only the first exon sequence of the alignment is shown but the entire expected protein coding sequence is present across the five exons. H. Multiple sequence alignment of TTG2 homologs, including *C. pallidicaule*, *C. suecicum*, the sub-genome B copies of *C. quinoa* and *C. berlandieri nuttaliae*, and the presumptive sub-genome B copy of the *de novo* assembly (scaffold27337). I. Predicted conserved functional domains for the *de novo* assembly scaffold27337-encoded spliced translation product.

References

1. Simon, S. FastQC: A Quality Control Tool for High Throughput Sequence Data [Online]. **2010**, http://www.bioinformatics.babraham.ac.uk/projects/fastqc/.
2. Kokot, M.; Dlugosz, M.; Deorowicz, S. KMC 3: counting and manipulating k-mer statistics. *Bioinformatics* **2017**, *33*, 2759-2761, doi:10.1093/bioinformatics/btx304.
3. Vurture, G.W.; Sedlazeck, F.J.; Nattestad, M.; Underwood, C.J.; Fang, H.; Gurtowski, J.; Schatz, M.C. GenomeScope: fast reference-free genome profiling from short reads. *Bioinformatics* **2017**, *33*, 2202-2204, doi:10.1093/bioinformatics/btx153.
4. Chen, S.; Zhou, Y.; Chen, Y.; Gu, J. fastp: an ultra-fast all-in-one FASTQ preprocessor. *Bioinformatics* **2018**, *34*, i884-i890, doi:10.1093/bioinformatics/bty560.
5. Kajitani, R.; Toshimoto, K.; Noguchi, H.; Toyoda, A.; Ogura, Y.; Okuno, M.; Yabana, M.; Harada, M.; Nagayasu, E.; Maruyama, H.; et al. Efficient de novo assembly of highly heterozygous genomes from whole-genome shotgun short reads. *Genome Res* **2014**, *24*, 1384-1395, doi:10.1101/gr.170720.113.
6. Jackman, S.D.; Vandervalk, B.P.; Mohamadi, H.; Chu, J.; Yeo, S.; Hammond, S.A.; Jahesh, G.; Khan, H.; Coombe, L.; Warren, R.L.; et al. ABySS 2.0: resource-efficient assembly of large genomes using a Bloom filter. *Genome Res* **2017**, *27*, 768-777, doi:10.1101/gr.214346.116.
7. Gurevich, A.; Saveliev, V.; Vyahhi, N.; Tesler, G. QUAST: quality assessment tool for genome assemblies. *Bioinformatics* **2013**, *29*, 1072-1075, doi:10.1093/bioinformatics/btt086.
8. Smit, A.F.A., Hubley, R, Green, P. . RepeatMasker Open-4.0. **2015**, http://www.repeatmasker.org.
9. Luo, X.; Chen, S.; Zhang, Y. PlantRep: a database of plant repetitive elements. *Plant Cell Rep* **2022**, *41*, 1163-1166, doi:10.1007/s00299-021-02817-y.
10. Simao, F.A.; Waterhouse, R.M.; Ioannidis, P.; Kriventseva, E.V.; Zdobnov, E.M. BUSCO: assessing genome assembly and annotation completeness with single-copy orthologs. *Bioinformatics* **2015**, *31*, 3210-3212, doi:10.1093/bioinformatics/btv351.
11. Tarasov, A.; Vilella, A.J.; Cuppen, E.; Nijman, I.J.; Prins, P. Sambamba: fast processing of NGS alignment formats. *Bioinformatics* **2015**, *31*, 2032-2034, doi:10.1093/bioinformatics/btv098.
12. Li, H. Exploring single-sample SNP and INDEL calling with whole-genome de novo assembly. *Bioinformatics* **2012**, *28*, 1838-1844, doi:10.1093/bioinformatics/bts280.
13. Danecek, P.; Bonfield, J.K.; Liddle, J.; Marshall, J.; Ohan, V.; Pollard, M.O.; Whitwham, A.; Keane, T.; McCarthy, S.A.; Davies, R.M.; et al. Twelve years of SAMtools and BCFtools. *Gigascience* **2021**, *10*, doi:10.1093/gigascience/giab008.
14. Lefouili, M.; Nam, K. The evaluation of Bcftools mpileup and GATK HaplotypeCaller for variant calling in non-human species. *Sci Rep* **2022**, *12*, 11331, doi:10.1038/s41598-022-15563-2.
15. Neph, S.; Kuehn, M.S.; Reynolds, A.P.; Haugen, E.; Thurman, R.E.; Johnson, A.K.; Rynes, E.; Maurano, M.T.; Vierstra, J.; Thomas, S.; et al. BEDOPS: high-performance genomic feature operations. *Bioinformatics* **2012**, *28*, 1919-1920, doi:10.1093/bioinformatics/bts277.
16. Quinlan, A.R.; Hall, I.M. BEDTools: a flexible suite of utilities for comparing genomic features. *Bioinformatics* **2010**, *26*, 841-842, doi:10.1093/bioinformatics/btq033.
17. Cabanettes, F.; Klopp, C. D-GENIES: dot plot large genomes in an interactive, efficient and simple way. *PeerJ* **2018**, *6*, e4958, doi:10.7717/peerj.4958.
18. Kapustin, Y.; Souvorov, A.; Tatusova, T.; Lipman, D. Splign: algorithms for computing spliced alignments with identification of paralogs. *Biol Direct* **2008**, *3*, 20, doi:10.1186/1745-6150-3-20.
19. Florea, L.; Hartzell, G.; Zhang, Z.; Rubin, G.M.; Miller, W. A computer program for aligning a cDNA sequence with a genomic DNA sequence. *Genome Res* **1998**, *8*, 967-974, doi:10.1101/gr.8.9.967.

20. Wu, T.D.; Watanabe, C.K. GMAP: a genomic mapping and alignment program for mRNA and EST sequences. *Bioinformatics* **2005**, *21*, 1859-1875, doi:10.1093/bioinformatics/bti310.
